# Jointly-hic: joint decomposition of contact frequency maps captures salient features of genome architecture across tissues and development

**DOI:** 10.1186/s13059-026-04067-1

**Published:** 2026-05-02

**Authors:** Thomas Reimonn, Vedat O. Yilmaz, Hoang Tran, Garrett Ng, Derek Liu, Nezar Abdennur

**Affiliations:** 1 Department of Genomics and Computational Biology, UMass Chan Medical School, Worcester, MA USA; 2 Department of Systems Biology, UMass Chan Medical School, Worcester, MA USA

**Keywords:** Compartmentalization, Genome architecture, Chromosome organization, Hi-C, Long-range interactions, Heterochromatin, Development

## Abstract

Chromosome conformation capture methods, such as Hi-C, have been used to profile chromosome organization from a wide variety of biosamples and conditions; however, existing methods for analyzing such datasets have disadvantages for large-scale integrative studies of long-range interactions. To address this shortcoming, we introduce an analytical framework, *jointly-hic,* that computes harmonized projections across arbitrarily many contact frequency matrices, suitable for integrative studies of compartmentalization and long-range interactions. Our approach produces robust and directly comparable first and higher-order principal component scores that collectively capture biologically meaningful information beyond traditional A/B compartment scores.

**Supplementary Information:**

The online version contains supplementary material available at 10.1186/s13059-026-04067-1.

## Background

Chromosome organization plays a pivotal role in cellular processes, including gene regulation, organ development, and cell identity determination [1–3]. The principles that shape genome architecture are crucial for facilitating physical proximity between regulatory elements and target genes, as well as replicating and maintaining chromosomes, and transferring them during cell division [4, 5]. The evolution of chromatin conformation capture-based molecular assays (e.g. Hi-C, Micro-C) has enabled comprehensive profiling of contact frequency maps of many biospecimens and conditions at multiple resolution scales [6–8]. Over the last two decades, these methods have revealed some of the major biophysical processes shaping genome architecture [9]. The first of these, loop extrusion by ATP-dependent SMC complexes, influences local genome organization in *cis* during interphase, giving rise to the patterns in contact maps collectively associated with Topologically Associating Domains (TADs) in vertebrates [10, 11]. The second major process, termed *compartmentalization*, is mechanistically independent of loop extrusion and gives rise to a genome-wide checkering pattern between chromosome domains within the same and across different chromosomes [12–14].

The checkering pattern seen in most interphase Hi-C maps is usually characterized as a binary partition of two groups of loci whose members interact preferentially with one another. In mammalian cells, these two groups or “compartments” are referred to as A and B and broadly correspond to transcriptionally active and inactive loci, respectively. The conventional analysis of genome compartmentalization consists of the calculation of the leading eigenvector of a suitably pre-processed normalized contact matrix or its correlation matrix, obtained from either intra- or inter-chromosomal maps [13, 15, 16]. The mathematical reason this one-dimensional profile is taken to define A/B compartments is because its outer product produces the checkerboard-like matrix of rank 1 that best reconstructs the input matrix in a least-squares sense [17–19].

**Fig. 1:**
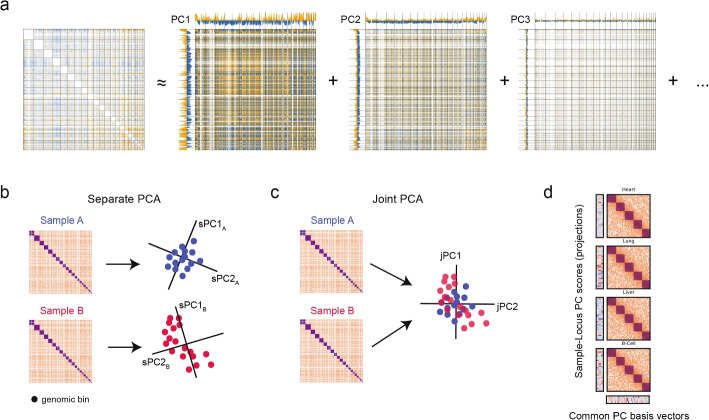
Overview of joint PCA for harmonized dimensionality reduction of multiple Hi-C datasets. **A** Schematic of the factorization of a genome-wide contact frequency map (or any derived similarity matrix) by a truncated eigendecomposition following mean centering. The sum of the eigenvalue-weighted outer products of the first n eigenvectors (or PC score vectors) gives the best rank-n reconstruction of the input matrix in terms of overall squared error. Traditional A/B compartment analysis corresponds to calculating only the first such vector from a single contact matrix. **B** Cartoon depicting applying PCA to two Hi-C datasets separately (sPCA), yielding unaligned PC basis vectors (depicted as coordinate axes, only first two shown) and PC projections (depicted by blue and red points) spanning distinct vector spaces. Note that, as a consequence of the unusual property of the sPCA inputs being square-symmetric, each sPC basis vector and the corresponding sPC score vector of the dataset’s projection happen to be numerically identical. This is not the case for joint PCA. **C** Depiction of applying joint PCA on two Hi-C datasets (jPCA) to produce a common set of n PC basis vectors spanning a common vector space (coordinate axes, only first two shown). Each of the two sample datasets (blue and red points) are projected onto this common coordinate system to produce n PC score vectors per dataset. **D** Depiction of a joint PCA of multiple Hi-C datasets as performed by jointly-hic that produces sample-specific projections (i.e. PC scores, left heatmaps) onto a common set of PC basis vectors (bottom heatmap) across all input samples. Each sample-locus pair is thus associated with a comparable n-dimensional compressed representation of its overall contact frequency profile

Studies using higher-resolution contact maps have long indicated that the A/B delineation and the continuous rank-1 approximation provide only a rough assessment of the complex patterns of long-range interactions [20, 21]. The partitioning of genomic loci into greater than two groups based on long-range contact frequency profiles is often referred to as identifying “sub-compartments”, though we have proposed Interaction Profile Groups (IPGs) as a more neutral, less suggestive term. The first methodologies for IPG identification applied unsupervised clustering directly on interchromosomal contact matrices [20, 22] and other heuristic approaches have been developed for A/B compartment and IPG identification from intrachromosomal contact matrices [23, 24]. Polymer simulations based on mechanistic biophysical principles as well as constraint-based and generative modeling approaches based on mechanistically driven assumptions have subsequently provided insight into the drivers of compartmentalization [25–28]. Unsupervised dimensionality reduction provides an essential complement to mechanistic modeling, by enabling the appraisal of the full scope of long-range interactions and their variability across cell types and conditions, as well as distinguishing biological from technical sources of variation. In fact, the traditional matrix decomposition approach to quantifying A/B compartments extends naturally to approximations of higher matrix rank (Fig. 1A). By generating additional component vectors, the reconstruction of the input matrix is improved, thereby better explaining the variance in the data. We recently showed how unsupervised clustering on such higher-order representations provides a scalable method for elucidating IPGs in individual Hi-C samples [21].

Overall, the origins and details of compartmentalization and other global features of genome organization in the nucleus remain unclear. While specific biochemical affinities between chromatin states are thought to be a major driving mechanism of chromosome compartmentalization [9, 29], evidence is also growing for the importance of the association of certain regions of the genome with nuclear bodies (e.g., speckles, nucleolus) and tethering to the nuclear lamina for understanding spatial and functional genome organization [30]. Indeed, classic A/B compartment intervals tend to be distributed radially in the nucleus, with euchromatin typically located centrally and inactive or heterochromatin located towards the nuclear periphery. Additionally, because of the slow timescale of global compartmental organization in interphase, Hi-C maps can also be influenced by the configurations of chromosomes upon exit from mitosis [31–33].

Advancements in the resolution and production of contact frequency maps, including from large concerted efforts such as ENCODE and the 4D Nucleome Consortium, has led to a diversity of 3D genomic datasets across a range of human cell types and tissues [34–37]. These datasets provide opportunities for integrative analyses across diverse biological contexts. However, current analytical frameworks are not yet suitable for the integration of many heterogeneous samples. The standard approach is to perform matrix decomposition separately on each sample, which results in each sample being projected onto a different linear subspace (Fig. 1B). As a result, while the first component vector derived from each of two contact matrices might explain reasonably well the primary compartmentalization pattern in each sample, strictly speaking, those two vectors are not compatible for one-to-one comparison. Careful normalization and manual post-processing steps are required to guarantee sufficient compatibility of A/B scores to support differential analysis [38, 39]. Overall, this approach greatly limits what can be learned by integrating information from numerous contact frequency maps simultaneously.

Producing a *joint* projection from all input samples avoids the incompatibility introduced by separate decomposition and avoids introducing biases. However, the computational limitations for memory and compute often make such large-scale joint analyses infeasible. To directly address this methodological gap, we present an integrative analytic framework and Python toolkit, called *jointly-hic*, that computes joint decompositions of interchromosomal contact frequency profiles from multiple datasets simultaneously, situating projections from all chromosomes and samples in a unified vector space (Fig. 1C, D). The toolkit applies a mini-batch incremental principal component analysis (PCA) algorithm that scales to arbitrary input sizes without incurring additional memory costs [40]. We establish the effectiveness of our method to coherently project multiple Hi-C maps into a common space spanned by multiple biologically informative basis vectors. We applied *jointly-hic* to a diverse collection of 89 Hi-C datasets, encompassing primary cells, human ex vivo tissues, and in vitro differentiation models of heart, pancreas, and liver development [41, 42]. Leveraging this atlas, we identified distinct nuclear organization patterns distinguishing immune cells, in vitro*-*derived cells, and other mature tissue samples, characterized in part by differences in repressive histone modifications. Finally, by integrating joint projections with gene expression and chromatin accessibility profiles, we show that changes in higher-order principal component scores are predictive of differential gene expression and regulatory element activity beyond the traditional A/B compartment score. Collectively, our approach establishes a scalable and robust foundation for systematically investigating genome organization across diverse biological contexts in order to provide critical insights into the structural underpinnings of gene regulation and cellular identity.

## Results

### Joint PCA produces robust and coherent projections of long-range genomic contact frequencies from multiple samples

We developed a framework that performs a joint PCA of interchromosomal contact frequency profiles from multiple Hi-C datasets simultaneously (Fig. 1C, D). By focusing on interchromosomal contacts, our approach produces projections that are not only harmonized across samples but also across all chromosomes. We note that the framework may be extended to *cis* contact frequency maps on an individual chromosome basis with limitations (see Methods). Our implementation, available as a Python package called *jointly-hic* [43], provides pre-processing and joint projection starting from genome-wide contact frequency matrices. The toolkit provides additional utilities for clustering, visualization, and integration with other epigenetic profiling datasets such as RNA-seq, ATAC-seq, or ChIP-seq.

We begin by defining some terminology. The conventional matrix-based approaches to the dimensionality reduction of Hi-C data are mathematically equivalent to variants of PCA, treating the rows of the input matrix as observations or training examples (see Methods). From the matrix factorization perspective, one obtains a single collection of eigenvectors, whose weighted outer products sum to an approximation of the original matrix (Fig. 1A). From the PCA perspective, one obtains a set of principal components (PCs) or *basis vectors* over the space of input features that define a new orthogonal coordinate system and a set of PC *score vectors* corresponding to the *projections* of the input observations onto the basis vectors. In the special case of a single symmetric contact matrix as input, the features (columns) and observations (rows) are the same and consequently the basis and score vectors are numerically identical. As such, distinguishing between the concepts of basis and projection has not been relevant in contemporary studies of compartmentalization. However, for a joint PCA trained on more than one input contact matrix, the distinction must be made explicitly. The principal component basis vectors are of cardinality the number of genomic bins or loci (features) and define a common set of coordinate axes in which to project the observations (Fig. 1C). Each observation, a row of an input sample contact matrix, corresponds to the interaction profile of what we term a *sample-locus*, and is associated with projections defined by scores along each basis vector. Therefore, each input sample matrix is associated with its own set of PC score vectors, one for each corresponding basis vector (Fig. 1D). Since we seek a minority of basis vectors that explain the most variance, the linear projections of sample-locus interaction profiles map to a lower-dimensional feature space, so we may also consider them embeddings.

We first sought to evaluate the robustness of our joint PCA approach to variation in data quality. To do this, we started with a deeply sequenced Hi-C dataset from the HCT116 human cell line and introduced synthetic perturbations [44]. Starting from ~ 2.23 billion valid pairs, we progressively downsampled the dataset by up to 90% (~ 223 million pairs) to simulate varying sequencing depths. We also replenished the contacts removed by adding progressively increasing levels of random ligation noise, up to 90% of pairs, to simulate poor library quality. Despite the data perturbations, comparisons of scatter plots of PC1 vs PC2 scores and the correlation map between PC score vectors demonstrate preservation of structure across a range of sequencing depths and background ligation noise levels (Additional file 1: Fig. S1A, B). These results confirm that joint decomposition integrates Hi-C data across a broad depth and quality spectrum in a robust and consistent manner.

**Fig. 2:**
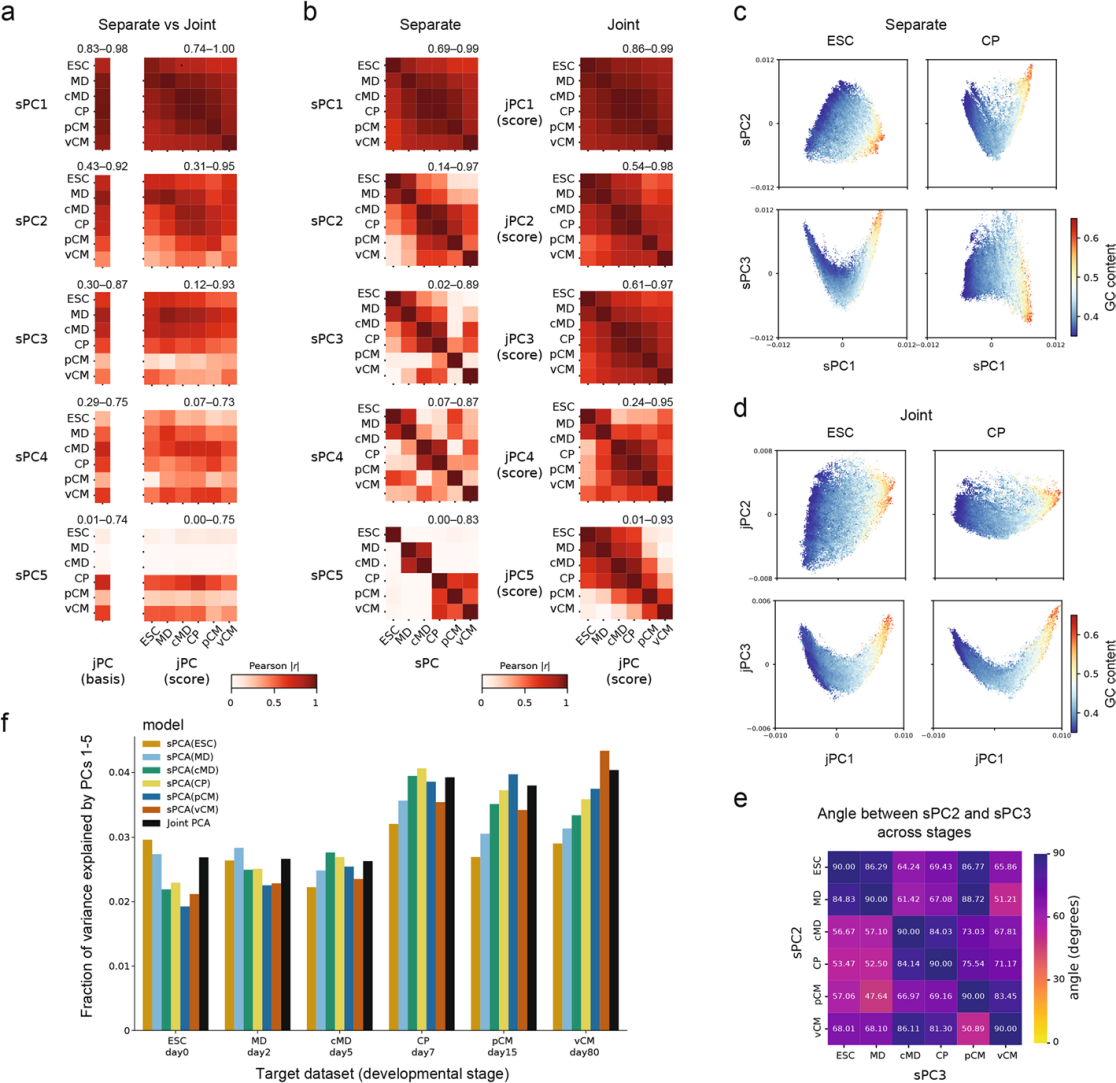
Joint PCA produces accurate and coherent projections of multiple samples onto a common set of basis vectors. Separate and joint PCA were performed on samples representing six successive in vitro cardiomyocyte differentiation stages labeled ESC, MD, cMD, CP, pCM, and vCM. **A** Left: absolute Pearson correlation coefficients between stage-specific sPC vectors and jPC common basis vectors. Right: absolute Pearson correlation coefficients between stage-specific sPC vectors and jPC score vectors for each stage. Value ranges (min–max) are indicated above each heatmap. **B** Left: cross-stage correlations between sPC vectors. Right: cross-stage correlations between jPC score vectors. Value ranges (min–max, excluding diagonal entries) are indicated above each heatmap. **C**,** D** Scatter plots of PC scores along the PC1 vs PC2 (top) and PC1 vs PC3 (bottom) basis vectors at the embryonic stem cell (ESC, left) and cardiac progenitor (CP, right) stages obtained by separate (C) and joint (D) PCA decompositions. Points, representing 50-kb bins, are colored by average GC content. These plots illustrate what appears qualitatively as a transposition of the geometry of the sPCA point clouds between stages due to changes in the directions spanned by sPC2 and sPC3. By contrast, geometries in the jPCA scatter plots remain consistent since each sample shares the same jPC2 and jPC3 basis vectors. **E** Heatmap of acute angles, in degrees, between sPC2 and sPC3 vectors across differentiation stages demonstrate that sPC basis vectors can deviate significantly from orthogonality between stages. Note that the common jPC basis vectors are all mutually orthogonal by construction. **F** Bar chart showing the quality of reconstruction of each stage’s pre-reduction input matrix after projection onto the first five PC basis vectors from each of the stage-specific sPCA models and the joint PCA model, as measured by the fraction of variance explained

We next sought to explore the mutual compatibility of jointly-derived (jPCA) versus separately-derived (sPCA) principal component projections from multiple samples for comparative analysis across biological conditions. For this purpose, we considered a sequence of six developmental stages from a published in vitro cardiomyocyte differentiation study: embryonic stem cells (ESC), mesoderm (MD), cardiac mesoderm (cMD), cardiac progenitor (CP), primitive cardiomyocyte (pCM), and ventricular cardiomyocyte stages (vCM) [42]. For each of the 50-kb pre-processed contact matrices corresponding to a differentiation stage, we performed both PCA on each stage’s matrix separately and on all stages' matrices jointly. We assessed the relationships between separately-derived vectors (sPC)—which serve as both basis and score vectors—and jointly-derived (jPC) basis and score vectors by calculating absolute Pearson correlations and by inspecting scatter plots of genomic bin PC scores (Fig. 2A, B, Additional file 1: Fig. S2). We expected that both separate and joint PC1 basis vectors would generally explain A/B compartmentalization, as this is the dominant source of pattern variation in virtually all mammalian interphase maps and, despite cell type-specific variation, is generally robust and universal. Accordingly, both sPC1 and jPC1 basis vectors track GC content in all six stages (Additional file 1: Fig. S2A, D, E) and sPC1 vectors are strongly correlated with both jPC1 basis (*r* = 0.83–0.98) and score vectors (*r* = 0.74–1.00) (Fig. 2A).

In contrast to PC1, we see that higher-order sPC vectors behave inconsistently: for example, sPC2 and sPC3 show weaker cross-stage correlations (Fig. 2B), and their shifting biological interpretations appear in the changing geometry of the scatter plots (Fig. 2C, D, Additional file 1**:** Fig. S2B-G). In early stages (ESC and DE), sPC2 captures distance from the centromere, but this relationship is diminished or lost in later stages (Additional file 1: Fig. S2A, F), illustrating a drawback of performing PCA separately, namely that basis vectors of the same rank across different samples need not represent comparable sources of variation. Moreover, sPCs of a given rank in one stage may correlate more strongly with sPCs of a different rank in another stage. This is exemplified by the inversion of point-cloud geometry: in ESC, the sPC1–sPC2 manifold is “sail-shaped” and sPC1–sPC3 is “crescent-shaped” but from cMD onward this relationship qualitatively flips (Fig. 2C, D, Additional file 1: Fig. S2B-G). This is further reflected in the angle between sPC2 and sPC3 basis vectors dropping as low as ~ 47.6° across stages (Fig. 2E), indicating cross-stage linear dependence rather than orthogonality. By contrast, jPC score vectors consistently exhibit stronger cross-stage correlation for PC1 and higher-order components (Fig. 2B), and the jPC2 basis vector captures distance from the centromere across all cardiomyocyte differentiation stages (Additional file 1: Fig. S2A, G).

Conceptually, jPC basis vectors derived from multiple contact maps produce an orthonormal coordinate system whose directions constitute a consensus that maximizes variance across all loci in the entire collection. As such, jPC basis vectors represent a compromise shaped by the structure of variation across samples [45]. This is evident when comparing the fraction of variance of the pre-reduction contact matrices explained by the basis vectors of each sPCA model and the jPCA model: the stage-specific models explain the most variance for their corresponding stage by a small margin (Fig. 2F). However, the joint model roughly matches or exceeds the performance of all other stage-specific models, suggesting that most sources of biological variation are preserved by jPCA of a given rank compared to sPCA (with additional information recoverable from higher jPC ranks). Overall, our results suggest that while sPCA and jPCA basis vectors span similar subspaces of contact feature space, in sPCA the basis vectors can be completely misaligned from sample to sample, making, for example, sPC2 from embryonic stem cells more closely aligned with sPC3 than with than with sPC2 from cardiomyocytes and vice versa. On the other hand, jPCA ensures by design that higher-order PC scores are always comparable between samples.

Our results further revealed that comparing samples using sPCA is problematic for another reason besides misalignment: the indeterminate signs of sPC basis vectors derived from different samples. Eigenvectors such as principal component basis vectors are unique only up to algebraic sign and the sign of a vector calculated by an eigensolver is arbitrary. Therefore, both to impart biological meaning as well as to compare different samples, a method or convention can be used to assign a sign or “orientation” deterministically. In conventional A/B compartment analysis, GC-content serves (in mammalian cells, at least) as a reference signal for choosing the orientation of the basis/score vector, such that the positive phase has higher GC content than the negative phase. Unless a similar orthogonal reference signal exists that predicts a given basis vector with high fidelity, no guidance exists to harmonize the signs of higher-order sPC vectors, since their indeterminate signs are independent of one another. Because joint PCA produces scores projected onto a common coordinate system, even though the signs of the jPC basis vectors are indeterminate, the projections (scores) from different samples always align with the common basis vectors and are therefore comparable.

Finally, we confirmed that jPCA is robust to varying data resolutions and that our *jointly-hic* implementation is computationally scalable. PC score vectors for the six stages across resolutions from 25 to 500 kb were highly correlated with those obtained at 100 kb (Additional file 1: Fig. S1C). Importantly, while computational demands scale with the inverse square of resolution, memory usage remains fixed relative to the number of input samples (Additional file 1: Fig. S3, Additional file 2: Table S1), ensuring that the joint projection remains stable and scalable for large, high-resolution atlases.

Taken together, our findings demonstrate that while sPCA may approximate global structure within individually analyzed samples, it fails to produce basis vectors suitable for comparison across samples. When comparing contact maps in detail across multiple biological conditions, a joint PCA approach is required. Overall, joint PCA provides robust, interpretable, and directly comparable projections across samples for comparative genome compartmentalization analysis.

### An atlas of long-range interaction profiles reveals distinct nuclear architectures associated with cell state

**Fig. 3:**
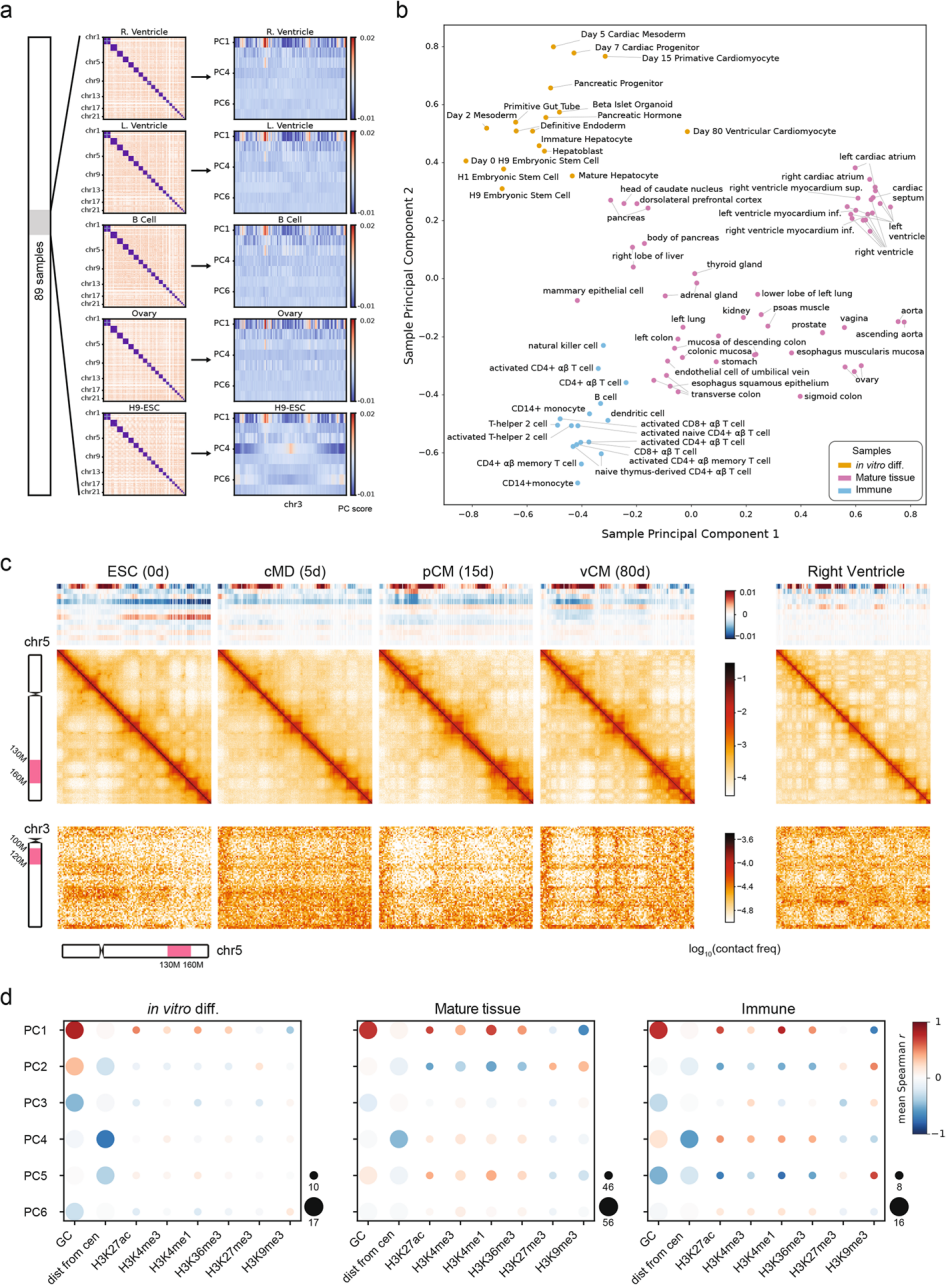
A locus-based atlas of long-range chromatin interaction profiles at 50-kb resolution across tissues reveals distinct genome architectural signatures between cell state categories.** A** Schematic of applying jointly-hic to 89 Hi-C datasets, yielding genome-wide principal components and PC scores at 50-kb resolution. Heatmaps of the first 7 PC score vectors for chromosome 3 shown as an example. **B** Overview plot of the sample similarity of long-range interactions via secondary PCA. Samples were projected onto the first two components of a secondary sample-level dimensionality reduction based on the first 10 PC score vectors from Hi-C. Points are labeled by biosample identifier and colored by cell state category. **C** PC projections and contact frequency maps obtained from four stages (embryonic stem cell, cardiac mesoderm, primitive cardiomyocytes, and ventricular cardiomyocytes) along an in vitro cardiomyocyte differentiation trajectory (left) and a cardiomyocyte-rich ex vivo heart right ventricle sample (right). Top row: Heatmap of PC score vectors 1–12. Middle row: cis contact frequency maps showing a 30-Mb region on chromosome 5. Bottom row: trans contact frequency maps showing the same region against a 20-Mb region on chromosome 3. **D** Bubble plots depicting mean Spearman correlation coefficients between the first six principal component score vectors and biosample-matching genomic features for samples grouped into cell state categories in vitro differentiation (left), mature tissue (middle), and immune cells (right). The size of each bubble depicts the number of PC score vector + genomic track pairs contributing to the statistic

With a scalable and robust framework to embed long-range chromatin interaction profiles across biological contexts, we decided to apply *jointly-hic* to explore long-range autosomal interactions across diverse healthy human tissues and model organ development systems. We selected 89 high-quality Hi-C datasets from the ENCODE project, 4D Nucleome Consortium, and other published sources. These datasets include ex vivo mature human tissues, primary immune cells, and in vitro differentiation models simulating liver, heart, and pancreas development [41, 42]. We applied stringent selection criteria for ENCODE datasets, requiring over 1 billion ligation pairs and more than 200 million non-negative pixels. Differentiation samples from the in vitro models were included irrespective of pair count. We generated jPCA projections of autosomal contacts for all samples at a 50-kb resolution, starting from *cis-*masked, *trans-*contact frequency matrices, producing 32 principal component vectors for each sample within a shared vector space (Fig. 3A, Additional file 1: Fig. S4A). Heatmaps of the PC scores reveal contrasting profiles between samples, not only in PC1 but higher-order PCs as well, suggesting that higher-order PCs capture biosample-specific differences in genome architecture beyond traditional A/B compartmentalization.

To help interpret the biological relevance of our Hi-C-derived PC projections, for each sample we concatenated PC score vectors for all components across all genomic bins and conducted a secondary dimensionality reduction to compare the overall similarity of samples. Similar tissues and cell types appeared to cluster in this sample-level projection (Fig. 3B). For example, cardiac tissues (heart ventricle, atrium, myocardium) cluster together, as do immune cells (CD4 +, CD8 +) (Fig. 3B). Moreover, hierarchical clustering of the sample-level interaction profiles grouped the samples into three high-level categories reflecting cell state: (i) H1 or H9 human embryonic stem cells and derived cells from in vitro differentiation experiments, (ii) mature immune cell samples, and (iii) other mature ex vivo adult tissue cell samples (Additional file 1: Fig. S4B).

Two additional mature samples grown in culture—mammary epithelial cells and umbilical vein endothelial cells—clustered with other in vitro samples, while localizing closer to immune samples in the sample overview PCA map, suggesting an effect of sample isolation conditions or sample clonality on long range interaction profiles (Fig. 3B). To assess the impact of other potential technical and biological covariates, we examined batch effects and donor characteristics within ENCODE samples (Additional file 1: Fig. S4C-F). While laboratory and assay methodologies exhibited some separation, samples predominantly localized with other samples of similar tissue type. Donor age also displayed some gradient effects, but these often coincided with tissue types reflecting varying ease of sample acquisition (e.g., peripheral blood vs heart or brain).

Importantly, while points representing in vitro differentiation samples appear near one another in the sample-level projection, contact maps from later stages of differentiation exhibited compartmentalization patterns progressively similar to their mature counterparts (Fig. 3B, C). These progressions were reflected in the sample-level projection, where points representing in vitro*-*derived mature hepatocytes approach those representing human liver samples, ventricular cardiomyocytes cultured at 80 days approach heart ventricle samples, and endocrine pancreas samples approach mature pancreas samples (Fig. 3B). The progressions were visible in the original data by comparing, for example, both *cis* and *trans* contact frequency maps of the cultured cardiomyocytes to those of a mature right ventricle tissue sample (Fig. 3C). We observed a trend toward the strong compartmental checkering pattern seen in the mature tissue sample, especially in the 80-day cultured sample.

To begin interrogating what type of information each component may be captured in our atlas, we grouped samples by cell state categories – in vitro differentiation, mature tissue, and immune – and for each category calculated the correlation of individual PC score vectors with genomic variables and epigenomic features where matching data was available (Fig. 3D). As expected, we find that PC1 scores correlate with GC content across all samples and cell state categories and exhibit positive correlation with active histone marks (H3K27ac, H3K4me3, H3K36me3) and negative correlation with repressive or silencing ones (H3K27me3, H3K9me3) (Fig. 3D). We find that across the three cell state categories, PC2 and PC3 scores exhibit inverse patterns of correlation with some features, including H3K27me3 signal. PC4 scores consistently exhibit a strong negative correlation with genomic distance from the centromere, suggesting that PC4 captures broad patterns of association between chromosome arms and centromere and telomere clustering in *trans* [13, 21, 22]. Note that this differs from the joint decomposition of the cardiac differentiation samples alone, where the chromosome arm signature was seen in PC2 (Additional file 1: Fig. S2A). Intriguingly, the correlation profile of PC5 in the mature tissue category is the inverse of that in the immune category, while appearing weak across the board in the in vitro category, suggesting that PC5 scores are discriminative of the three categories of cell states (Fig. 3D). In the following section, we find evidence for other atlas basis vectors that discriminate between cell state categories.

Collectively, our results suggest that broad long-range contact frequency signatures as encoded using our joint PCA approach reflect cellular identity and capture overall biospecimen similarity and functional differences between samples. Furthermore, our analysis at the sample level shows that joint PCA can discriminate between two categories of mature cells and between immature and mature cellular states, suggesting that samples within these cell state categories possess different baseline nuclear architectures.

### Long-range interactions distinguish broad chromatin states within and across cell state categories

To explore the collective interaction manifold more comprehensively at the individual locus level, we applied unsupervised clustering to the joint PC projections in two ways. First, we considered the interaction profile of each individual locus in the context of its sample, thus taking the same genomic locus in two different samples as distinct observations. Conventional subcompartment/IPG detection involves the unsupervised clustering of long range contacts from a single contact matrix at a time [20, 21]. For integrative analyses, matching cluster identities and resolving clustering ambiguities and interpretations across samples becomes increasingly unwieldy as the number of samples increases. To eliminate the need to perform ad hoc label correspondence across samples and other post-clustering harmonization measures, we instead take advantage of the common jPC projection space to detect clusters of similar interaction profiles across all sample-loci in our atlas simultaneously. Here, we refer to this approach as *sample-locus* (SL) clustering (see Methods).

The second approach, which we refer to as *ensemble-wide locus* (EL) clustering, focuses on each locus’s complete set of interaction profiles across all samples in the atlas, thus considering each 50-kb genomic locus as a distinct entity. The latter approach is expected to group genomic loci together based on their sharing broadly coherent long-range interaction signatures across all of the samples in the atlas. Hence, while SL clustering can discern different interaction patterns of a given locus in different samples, EL clusters will consolidate interaction patterns that may differ from sample to sample but are correlated (e.g., loci having PC scores of opposite signs in two different samples). In both scenarios – SL and EL clustering – the final numbers of clusters, 11 and 8, respectively, were manually chosen by balancing of interpretability and model complexity.

**Fig. 4:**
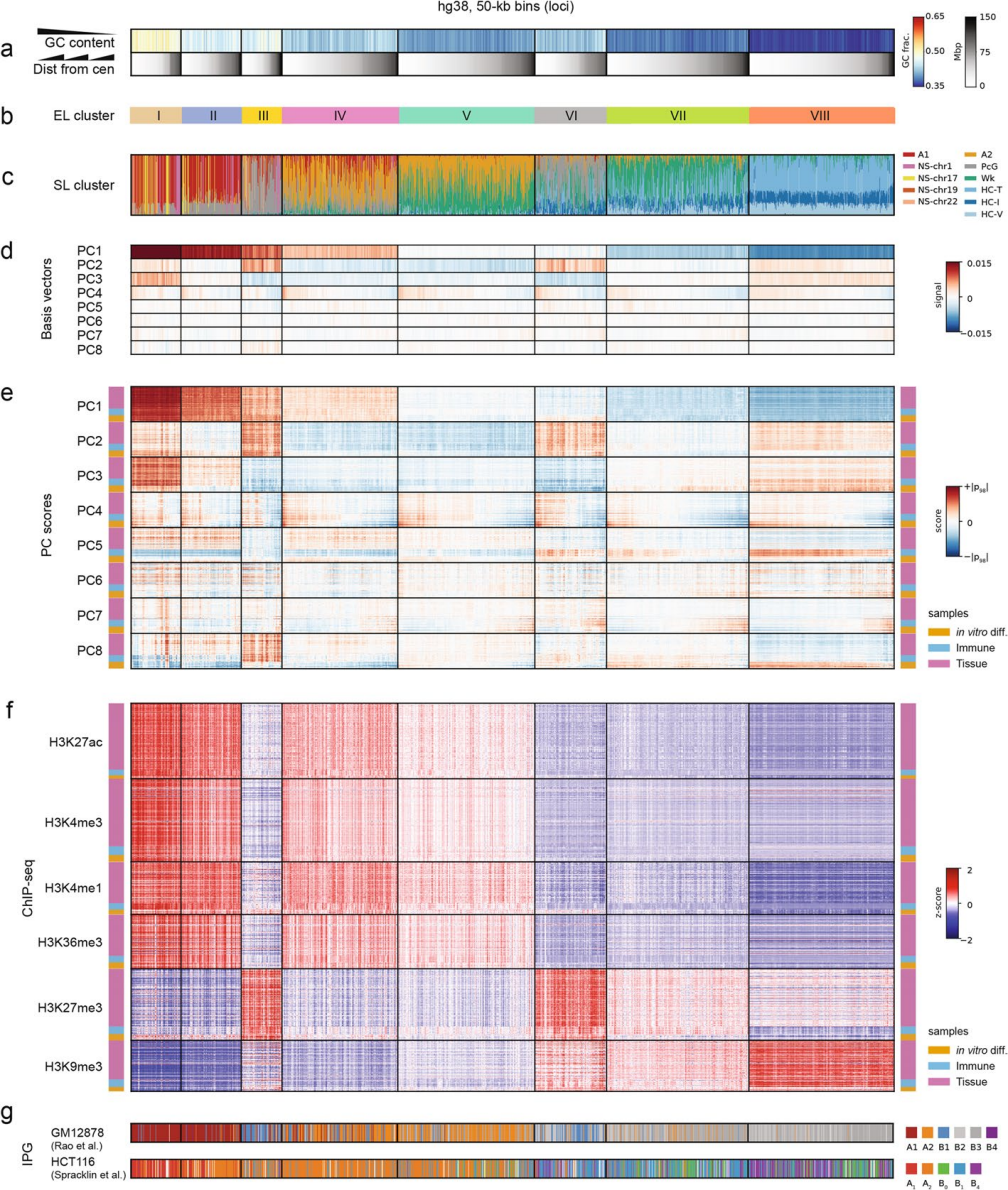
Locus-level cluster analysis and epigenetic characterization of the Hi-C interaction profile atlas. Multiple aligned heatmaps of binned genomic tracks characterizing all 50-kb loci (genomic bins) mapped across 89 Hi-C samples. Pixelwise, columns correspond to genomic loci and rows to different genomic data tracks. Columns (loci) are grouped into ensemble-wide locus (EL) clusters, separated by black vertical borders. The EL clusters (column groups) themselves are displayed in descending order of mean GC content. Within each EL cluster (column group), the loci (columns) are ordered by genomic distance from the respective chromosome’s centromere. The order of loci (columns) is the same across all heatmap panels. **A** Heatmaps of GC content (first row) and genomic distance from the centromere (second row) for all loci. These are the tracks used for column sorting. Colorbars are on the right. **B** Ensemble-wide locus (EL) cluster assignments corresponding to each column group labeled with Roman numerals. **C** Sample-locus (SL) cluster label frequencies for each locus. Each column is a stacked bar representation of the total number cluster label assignments of each type (colors) across all 89 Hi-C samples for the corresponding locus, ordered according to the color legend on the right. **D** Heatmap of PC basis vectors. Rows correspond to PCs 1–8. **E** Heatmap of PC scores for PCs 1–8 for each sample-locus in the 89 Hi-C samples. The heatmap is divided into eight row groups, each group corresponding to a PC rank. Within each row group the heatmap rows are ordered by cell state category as shown in the additional colored bars flanking the left and right sides of the heatmap. **F** Heatmap of z-score normalized ChIP-seq signals from ENCODE for biosamples matching those in the atlas. The heatmap is divided into six row groups, one corresponding to each of H3K27ac, H3K4me3, H3K4me1, H3K36me3, H3K27me3, and H3K9me3. Within each row group the rows are sorted by cell state category as shown in the colored bars flanking the left and right sides of the heatmap. **G** Interaction profile group (IPG) assignments for GM12878 (first row) and HCT116 (second row) cell lines from previously published studies. Legends for the categorical color labels are given on the right

To obtain a global overview of the two strategies, we visualized the results using a detailed locus-centric multi-panel heatmap, where each column corresponds to a 50-kb genomic bin (locus) and rows correspond to genomic feature tracks (Fig. 4). The columns are sorted into eight groups representing EL locus clusters, labeled using Roman numerals and depicted by the colored bars in Fig. 4B. The EL cluster groups themselves are ordered by descending mean GC content of the bins they contain, and within each EL cluster group the columns are ordered by ascending genomic distance from the centromere, as reflected in the tracks shown in Fig. 4A. The heatmap is organized into a series of panels displaying different feature categories. The first panel depicts for each locus the total frequencies of the eleven SL cluster labels across all samples as vertically stacked colored bars (Fig. 4C). The next panel displays the individual first eight PC basis vectors of the atlas (Fig. 4D). The subsequent panel arranges the corresponding PC score vectors from all 89 atlas samples (Fig. 4E). Scores corresponding to each PC rank appear in a unique row group, and within each PC rank row group, the rows are ordered according to the corresponding sample’s cell state category, as shown in the color legend bars to the left and right of the heatmap. The next panel arranges 1,410 ChIP-seq signal tracks for various histone marks derived from biosamples matching those used in the atlas, with one separate row group for each histone mark target, ordering the rows again by the sample of origin’s cell state category (Fig. 4F). Each row depicts the sample-wide z-score of ChIP-seq fold change over input signal for every 50-kb bin. Finally, we include two tracks displaying published IPG labels from previously characterized cell lines (Fig. 4G). To supplement the detailed heatmap with a visual aid, we also reduced the full projection of ~ 44,000 50-kb loci from all 89 samples in the atlas using Uniform Manifold Approximation and Projection (UMAP) [46], and colored the sample-locus points in the projection by each of the major groupings in Fig. 4: the corresponding sample’s cell state category (Additional file 1: Fig. S5A), the sample-locus’s SL cluster label (Additional file 1: Fig. S5B) and the corresponding locus’s EL cluster label across all samples (Additional file 1: Fig. S5C).

The detailed heatmap contextualizes the relationships between the Hi-C-derived PC scores, SL and EL cluster assignments, chromatin state, and cell state category. For example, consistent with Fig. 3D, PC4 scores display a strong negative gradient with distance from the centromere within every EL cluster, supporting that PC4 captures a universal signature of interactions along chromosome arms in *trans* (Fig. 4D, E). This gradient appears steepest in in vitro samples, perhaps influenced by higher cell division rates, shorter gap phases, and resulting differences in cell cycle composition. By contrast, PC7 exhibits a centromere-telomere gradient in in vitro and immune samples but not in mature tissue samples, and the gradient appears more pronounced in clusters with lower transcriptional activity (low PC1 score) (Fig. 4E). Components that discriminate cell state categories are also visible in the heatmap: PC2, PC3, and PC8 display unique score signatures in in vitro samples relative to the others, while PC5 shows an inverted signature in immune samples relative to in vitro and mature tissue samples (Fig. 4E).

At the sample-locus level, consistent with the characterization of the traditional A compartment, most SL clusters with positive PC1 score are enriched for classical active chromatin marks such as H3K4me3, H3K27ac, H3K36me3, and H3K4me1 (Additional file 1: Fig. S6A, C). The SL clusters with the highest PC1 score were also enriched for POL2RA ChIP-seq and SON TSA-seq in all available cell lines where these marks were assayed, indicative of nuclear speckle-association (Additional file 1: Fig. S6D, E). SL clusters that exhibited strong enrichment for active marks without enrichment for repressive marks were annotated, in accordance with precedent, A1 (active, speckle-enriched) and A2 (other active) according to activity levels and SON enrichment. Additionally, of the top six SL clusters by mean PC1 score, four of them were small clusters harboring dense, speckle-associated regions unique to chromosomes 1, 17, 19 and 22, respectively (Additional file 1: Fig. S6B), which appear as isolated “islands” via UMAP (Additional file 1: Fig. S6A).

Surprisingly, among sample-locus pairs having the lowest average PC1 scores, we found three separate SL clusters of classical B-compartment sample-loci—which we labeled HC-V, HC-T, and HC-I—each consisting primarily of sample-loci in vitro samples, mature tissue samples, and immune samples, respectively (Additional file 1: Fig. S5A, B, D). Despite selectivity for specific cell state categories, these three SL clusters share 60% of unique genomic loci in common (Additional file 1: Fig. S5E). Interestingly, loci with positive PC2 scores and negative PC3 scores were associated with elevated H3K27me3 levels. Often these features co-occur with PC1 scores at similar levels to A2 bins, forming a separate SL cluster. We labeled this cluster PcG, as the features are indicative of polycomb-repressive states within broader regions of elevated transcriptional activity (Fig. 4C, F, Additional file 1: Fig. S6C). Additionally, we observed a SL cluster of loci lacking H3K27me3 which tended to exhibit marks of activity at levels lower than loci in A2 and/or marks of repression lower than those of the HC-V, HC-T, and HC-I clusters (Fig. 4C, F, Additional file 1: Fig. S6C). We termed this latter cluster Wk for “weak”. The Wk loci are characterized by generally low histone modification signals for marks assayed by ENCODE but considerable variability depending on biosample context. To further examine regulatory activity across sample-loci in the atlas, we gathered ENCODE ATAC-seq data from mature tissue biosamples and estimated the most “active” regulatory elements as the top 100,000 (~ 4%) of candidate *cis-*regulatory elements (cCREs) [35] per biosample by ATAC-seq signal. We found that A1 and the nuclear speckle SL clusters have the highest density of active regulatory elements, closely followed by A2. PcG and Wk EL clusters have low active cCRE density and HC-T is nearly depleted of active cCREs (Additional file 1: Fig. S6F).

We compared the SL cluster assignments across all sample-loci in the atlas with the six-cluster subcompartment classifications of the same loci in GM12878 [20] and the five-cluster IPG classifications in HCT116 [21], and found general agreement in interpretation (Additional file 1: Fig. S5F, G). Sample-loci in the speckle island and A1 SL clusters correspond mostly to loci with the A1 subcompartment assignment in GM12878 and the A_1_ label in HCT116. Additionally, loci labeled B4 in GM12878, which are unique to chromosome 19, are uniquely found in the NS-chr19 island cluster. Note that this differs from B_4_ in HCT116, which is thought to reflect a similar underlying chromatin state to B4 in GM12878 but is distributed in multi-megabase domains throughout the genome in HCT116 [21]. Sample-loci in SL cluster A2 map mostly to GM12878 A2 loci and HCT116 A_2_ loci, and sample-loci in the PcG cluster map mostly to loci with the B1 subcompartment label in GM12878. By contrast, the B_1_ IPG label in HCT116, associated with dispersed regions of H3K27me3 flanking large B_4_ domains, was most enriched for sample-loci assigned the Wk cluster label. Sample-loci in the Wk cluster corresponded to GM12878 loci with a mixture of mostly A2 and B2/B3 assignments. Finally, sample-loci in our cell state-specific inactive clusters HC-V, HC-I and HC-T predominantly corresponded to B2/B3 loci in GM12878 and to B_0_, B_1_, and B_4_ loci in GM12878. We have previously observed that B2 and B3 appear to differ only in their centromere-telomere preference, and likely represent a single silent chromatin state in GM12878, while B_0_ and B_4_ were shown to correspond to broad H3K9me2 and H3K9me3/HP1 states, respectively, in HCT116 [21].

At the ensemble-wide level, the EL clusters were found to be associated with epigenetic states that are broadly similar across the biosamples in the atlas with a few exceptions (Fig. 4B, F). The first two EL clusters—I and II—are the most enriched for marks of transcriptional activity, encompassing most sample-loci with speckle-associated SL assignments. Clusters IV and V have progressively lower enrichments for active marks and comprise mostly sample-loci with the A2 and Wk SL cluster assignments. Interestingly, EL cluster VII consists of classical B-type loci that show only a weak enrichment for either of the two conventional heterochromatin marks (H3K27me3 or H3K9me3). Sample-loci with the Wk SL cluster assignment are primarily divided across between EL clusters V (weakly active) and VII (weakly repressive).

Interestingly, four EL clusters are strongly associated with the presence of conventional heterochromatin marks in a majority of samples (Fig. 4B, F). EL clusters III and VI are enriched for H3K27me3, exhibit high PC2 and low PC3 scores in most samples, and comprise most sample-loci with the PcG assignment. Notably, EL clusters III and VI differ in their predominant PC1 status, with cluster-III loci corresponding to classic A-type loci (positive PC1) and cluster-VI loci corresponding to classic B-type loci (negative PC1). Together, this indicates that the presence of H3K27me3 is associated with different interaction signatures depending on the broader functional genomic context. EL cluster VII is depleted for active marks and exhibits mild levels of both H3K27me3 and H3K9me3. Finally, cluster VIII comprises the majority of sample-loci harboring the three cell state-specific SL inactive or heterochromatic cluster assignments, HC-V, HC-I and HC-T, and is strongly enriched for H3K9me3.

To explore the relationship between PC projections and the proximity to nuclear landmarks, we gathered distance-dependent biotin labeling data from TSA-seq experiments in H1 hESC and HFFc6 cells [30, 46] as well as Spatial Position Inference of the Nuclear Genome (SPIN) states derived from a combination of TSA-seq, DamID-seq and *cis* Hi-C data [47] (Additional file 1: Fig. S7). Visually, the projection of H1 sample loci along PC1 and PC2 appeared to separate most SPIN state labels reasonably well (Additional file 1: Fig. S7B). This was confirmed by calculating silhouette score distributions for each SPIN label with respect to different two-dimensional projections. In PC1-PC2, all distributions were positive-skewed towards 1, indicating reasonable separation, with the exceptions of InteriorRepr1 and InteriorRepr2, which overlap one another, and InteriorAct2, which overlaps InteriorAct1 (Additional file 1: Fig. S7H). Together, this suggests that a significant amount of information about spatial positioning is contained in the first two components of *trans* Hi-C data alone.

As expected, we observed that Lamin B1 proximity was associated with the lowest PC1 scores, while SON proximity was associated with the highest PC1 scores (Additional file 1: Fig. S7B-D). SON was also associated with high PC2 and PC3 scores and enriched in loci corresponding to the nuclear speckle island SL clusters (Additional file 1: Fig. S7D, Fig. S6E). TSA-seq for centromere protein B (CENPB), which binds centromere-flanking alpha-satellite repeats, followed a similar pattern to SON TSA-seq (Additional file 1: Fig. S7D, G), consistent with previous observations of close proximity or contact of a fraction of centromeres to nuclear speckles in H1 cells [48]. Immunostaining of SON and NIFK/MKI67IP in the same study also suggested that nuclear speckles surround nucleoli in H1 cells [48]. Interestingly, in PC projections, TSA-seq for nucleolar markers differed slightly for MKI67IP, a marker of the Granular Component (GC) of nucleoli, and POLR1E, a subunit of RNA Polymerase I associated with the Follicular Centers (FCs) within nucleoli: while the signal from the FC marker was evenly distributed across PC3 levels, we found that the signal from the GC marker formed a shallow gradient along PC3 opposing that observed for SON (Additional file 1: Fig. S7D-F). We speculate that this difference may be due to the spatial localization of tyramide-radical sources within the nucleolus (Additional file 1: Fig. S7I). TSA-seq is expected to show more precise spatial labeling (steeper exponential decay) for markers of FCs, while markers for GCs, which occupy a larger fraction of the nucleolus, will exhibit more diffuse labeling [30, 48]. Since FCs are spatially localized throughout the nucleolus and act as compact point sources, chromatin positioned in the nucleolus and near the periphery (e.g., speckle-proximal chromatin) are expected to be labeled with comparable efficiency by an FC marker. Conversely, TSA-seq from a GC marker may show a bias, preferentially labeling chromatin in the interior over the periphery, leading to the observed gradient along PC3 (Additional file 1: Fig. S7F, I). Consequently, we propose that, for loci with high PC2 score, PC3 may be capturing a nucleolus-to-speckle axis for chromatin, at least in H1 cells.

### Cell state-specific differences are associated with the heterochromatic composition of a common set of loci

It was surprising that the same large collection of genomic loci (EL cluster VIII) separated into three distinct transcriptionally inactive SL cluster assignments that are unique to each cell state category (Additional file 1: Fig. S5D). Visually, these three sample-locus clusters diverged into three cell state-specific “lobes” in the UMAP embedding (Additional file 1: Fig. S5A). These observations prompted us to investigate whether compartmentalization differences in the predominantly silent portion of the genome may drive much of the global differences between the general architectures of these three categories of samples.

**Fig. 5:**
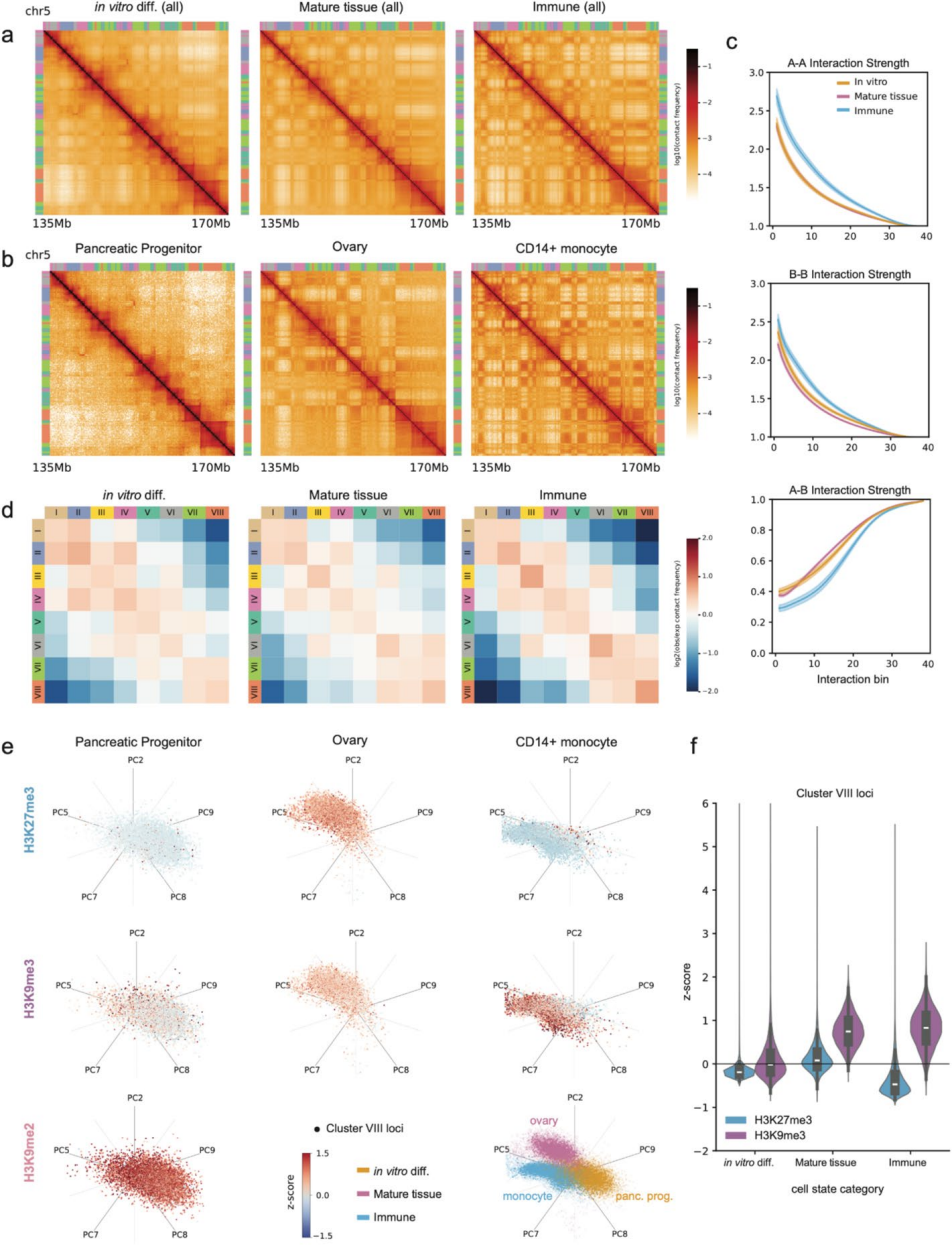
Samples in three cell state categories are associated with differences in the strength of heterochromatin compartmentalization and repressive histone mark composition. **A** Contact frequency maps generated from aggregating all samples within cell state categories show qualitative differences in average compartmentalization strength in *cis*. A 35-Mb region on chromosome 5 is shown. **B** Contact frequency maps for representative samples from each cell state category: pancreatic progenitor cells (in vitro differentiation), ovary (mature tissue), CD14 + monocytes (immune cells). **C** Interaction strength plots derived from intrachromosomal saddle plots based on PC1 score quantile. Curves represent the mean interaction strength in each cell state category with standard error envelope. **D** Discrete saddle plots of average observed over expected contact frequency between loci in EL clusters across all samples in each cell state category. **E** Star coordinate scatter plots projecting genomic loci comprising EL cluster VIII along PC2, PC5, PC7, PC8 and PC9 for each of the representative samples from the three cell state categories. Points are colored by z-score of ChIP-seq signal for repressive histone marks H3K27me3 (top row) and H3K9me3 (middle row) from the same biosample. In the bottom left, the average H3K9me2 ChIP-seq signal from H9 ESCs and H9-derived mesoderm, cardiomyocytes, liver progenitors, and hepatocytes is overlaid [49]. In the bottom right, the point clouds from each of the three representative samples are plotted together and colored by cell state category. **F** Violin plots of H3K27me3 and H3K9me3 ChIP-seq z-scores across matching biosamples in each cell state category

To observe the differences more directly, we aggregated all Hi-C datasets within each cell state category into pooled contact maps and visualized compartmentalization patterns (Fig. 5A). We found that the average immune cell map exhibited the strongest compartmentalization, characterized by prominent checkerboarding patterns, especially in EL cluster VII and VIII bins. The average mature tissue sample exhibited intermediate levels of compartmentalization of the same regions, with moderately defined checkering patterns, while in vitro differentiated samples displayed the weakest compartmentalization, characterized by smoother contact maps and less-defined genomic domain boundaries. We confirmed that these observations in the pooled maps were not averaging artifacts and were also evident in contact maps from individual samples from each category, such pancreatic progenitor cells (in vitro), ovary (mature tissue) and CD14-positive monocytes (immune) (Fig. 5B).

To quantify these observations systematically, we generated saddle plot heatmaps of observed-over-expected contact frequency, consolidating genomic bins into 40 ranked groups based on their joint PC1 score quantile (Additional file 1: Fig. S8A). As observed in the pooled contact maps, the composite saddle plot of immune samples showed significantly higher classic A-to-A and B-to-B interactions and notably weaker interactions between A and B regions than in vitro and mature tissue samples in *cis*. Profiling the homophilic and heterophilic interaction strengths from the diagonals of individual samples’ saddle plots confirmed that immune cells demonstrated the strongest compartmentalization with higher self-affinity for bins with similar PC1 scores and greater segregation of between bins with opposing PC1 scores, while in vitro and mature tissues shared similar interaction strength profiles with respect to PC1 (Fig. 5C). Additionally, we generated “discrete” saddle plot heatmaps of observed-over-expected contact frequency for each cell state category, grouping genomic bins into EL clusters as discrete categories (Fig. 5D, Additional file 1: Fig. S8B). These heatmaps broadly agreed with the PC1 saddle plots. The inactive EL clusters VI, VII, and VIII were depleted for interactions with other EL clusters and the depletion was more severe in immune samples. Clusters VI and VIII both exhibited stronger self-affinity in immune samples as well. Interestingly, cluster VI was found to have lower affinity for clusters VII and VIII in in vitro samples than in mature tissue and immune ones.

Comparing these results to the depiction of EL clusters VII and VIII in Fig. 4, we noticed differences in enrichment of marks H3K27me3 and H3K9me3 between cell state categories. In the case of EL cluster VII, H3K9me3 enrichment is relatively consistent across samples. However, while H3K27me3 is generally—albeit weakly—enriched in these loci for both tissue and immune samples, the same mark is generally depleted in the same loci in in vitro samples. In the case of cluster VIII, loci are enriched for H3K27me3 in mature tissue samples but depleted for the same mark in both immune and in vitro samples. Simultaneously, a strong enrichment for H3K9me3 is seen in tissue and immune samples yet not in in vitro samples. Given that PC basis vectors 2, 5, 7, 8 and 9 appeared to distinguish sample-loci by cell state category, we projected all the genomic loci in cluster VIII from characteristic samples from each category—pancreatic progenitor, ovary, and CD14 + monocyte—onto a multivariate star coordinates plot [50] (Fig. 5E). Coloring the points by sample confirmed a clear separation by cell state category, as expected. Splitting the points from each sample into separate plots, we then colored them by histone modification z-scores for H3K27me3 and H3K9me3, which supported the observation that cluster VIII loci have a mild to neutral enrichment of H3K27me3 in tissue samples, while being depleted for H3K27me3 in in vitro and immune samples. At the same time, these same loci are strongly enriched for H3K9me3 in tissue and immune samples but not in in vitro samples. Finally, the observed asymmetries in H3K27me3 and H3K9me3 levels in cluster VIII loci were confirmed by plotting distributions of sample-loci z-scores across the three cell state categories (Fig. 5F).

Interestingly, we found that for in vitro samples, genome-wide, broad H3K9me3 enrichment is largely limited to peri-centromeric, telomeric regions and characteristic domains on chromosome 19 (Additional file 1: Fig. S8C, D), while most of the peripheral, lamin-associated regions in the genome are enriched for H3K9me2 rather than H3K9me3 (Additional file 1: Fig. S8D-F, Additional file 1: Fig. S7C). Together, these results suggest that chromosome organization is influenced not by simple binary affinities between heterochromatic regions but are affected by combinatorial differences in the epigenetic composition of heterochromatic or pre-heterochromatic domains.

### Changes in jointly-derived PC scores coherently predict gene expression dynamics during in vitro development

**Fig. 6:**
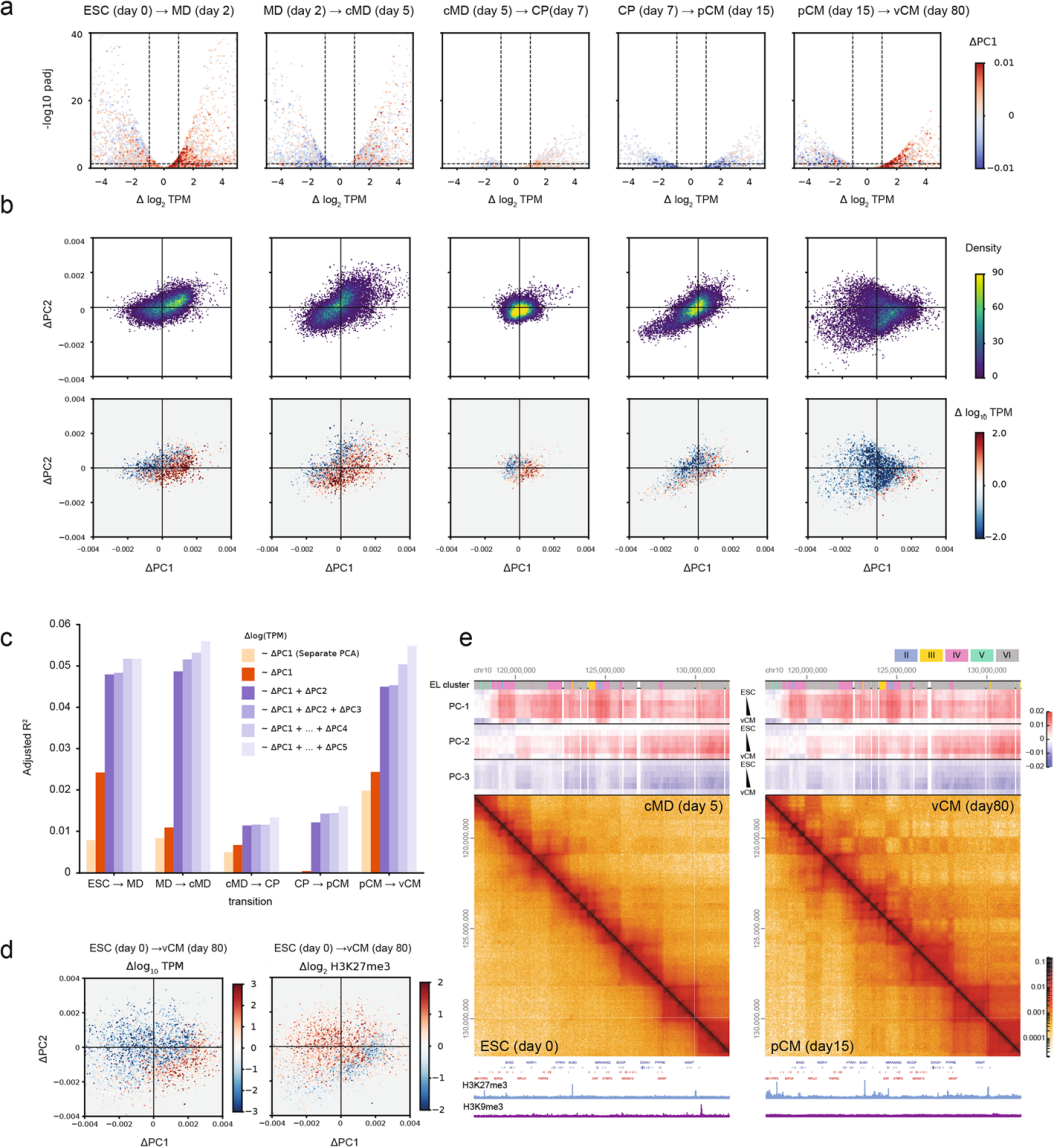
Atlas PC2 captures additional gene regulatory information during differentiation associated with repressive marks within active regions. **A** Volcano plots of differential gene expression between consecutive stages of cardiomyocyte differentiation, colored by change in PC1 score. **B** Scatter plots of change in PC1 score vs change in PC2 score for genes, colored by point density (top) and log fold change in gene expression (bottom). **C** Adjusted coefficients of determination for linear regressions predicting differential gene expression (log fold change in TPM) from differential PC scores for each stage transition. Models were trained on joint atlas PCs 1 through 5 cumulatively as well as for separately calculated PC1. **D** Scatter plots of change in PC1 score vs change in PC2 score for genes between the first (ESC) and last stage (ventricular cardiomyocytes) colored by log fold chane in gene expression (left) and log fold change in H3K27me3 ChIP-seq signal between H9 cells and in vitro RUES-derived cardiomyocytes from ENCODE. **E** HiGlass visualization of contact maps from four stages (left view: ESC, cMD, right view: pCM, vCM) centered on a 15-Mb region of chromosome 10. EL cluster labels and PC score tracks for all six differentiation stages are displayed on top of both views. Underneath both views are gene annotations and BigWig tracks of H3K27me3 and H3K9me3 from H9 (left view) and RUES-derived cardiomyocytes (right view)

Previous work has associated compartmentalization changes, as transitions between B compartment (negative PC1 scores) and A compartment (positive PC1 scores), with transcriptional activity shifts during cellular differentiation [51, 52]. To systematically investigate this relationship using our atlas, we turned again to the comprehensive six-stage in vitro cardiomyocyte differentiation dataset comprising matched Hi-C, RNA-seq, and ATAC-seq assays [42] and performed differential expression analysis between successive development stages. We colored volcano plots of differential gene expression by changes in jPC1, which revealed that genes with high expression changes tended to have high changes in jPC1 scores (Fig. 6A), consistent with the hypothesis that classic A/B compartmental status changes contribute to gene expression changes during development. However, the extent to which higher order principal components capture meaningful expression and regulatory changes remained unclear.

To determine whether higher-order PCs capture additional layers of regulatory information, we examined the relationship between changes in jPCA scores (ΔjPC) and changes in gene expression. Scatter plots of gene-level ΔjPC1 versus ΔjPC2, colored by log_10_ TPM fold change, showed clear diagonal gradients, indicating that a combination of ΔjPC1 and ΔjPC2 better explains transcriptional dynamics than ΔjPC1 alone (Fig. 6B). A similar trend was observed when coloring by changes in chromatin accessibility at ENCODE cCREs (Additional file 1: Fig. S9A, B). To quantify this information and examine the contribution of each PC to gene expression dynamics, we fit a series of linear regression models using ΔjPC1 through ΔjPC5 to predict log TPM fold change across each stage transition (Fig. 6C). As a baseline, we also included models using ΔsPC1 from separately-calculated PCA on each stage. Across all transitions, ΔjPC1 consistently outperformed ΔsPC1, as measured by adjusted R^2^, suggesting that the joint PC projection not only provides a biologically coherent coordinate system for comparative analysis, but one that better captures transcriptional changes in this dynamic experiment. Importantly, the inclusion of ΔjPC2 substantially improved model performance, explaining up to 5% of the variance in log TPM changes. We observed that performance increased only marginally beyond ΔjPC2, indicating that most of the predictive signal is captured by changes in the first two joint components.

The observation that changes in higher order components capture information relevant to gene expression dynamics independent of ΔPC1 is supported by our earlier observation that a pattern of PC2 and PC3 in the atlas are associated with the two EL clusters enriched for the repressive histone modification H3K27me3 across most samples (Fig. 4A-E). Namely, we observe that PC2 and PC3 take on anticorrelated positive and negative signs, respectively, in EL clusters III (H3K27me3-enriched with high PC1 score) and VI (H3K27me3-enriched with low PC1 score), while taking on similar values in the other quiescent EL clusters VII and VIII. This suggested that the combination of PC2 and PC3 can discriminate between different types of silencing chromatin states, such as Polycomb-repressed states within peripheral, gene-poor or interior, speckle-rich regions, and that the inclusion of PC2 and to some extent PC3 captures higher-order patterns of chromatin silencing than is possible using the traditional A/B compartment score. To test this, we collected H3K27me3 ChIP-seq data from ENCODE for H9 cells (the first stage) and for a different in vitro cardiomyocyte sample derived from RUES cells, as a proxy for the last stage. Coloring gene-level ΔjPC1 and ΔjPC2 for ES cells versus ventricular cardiomyocytes by log fold change in TPM and H3K27me3 showed inverted gradients along the same direction, where lower gene expression and increased H3K27me3 are associated with decreases in both jPC1 and jPC2 and vice versa (Fig. 6D). Supporting this, we could find examples of large regions of positive PC1 (classic A) and positive PC2 score in early differentiation stages, that fragment in later stages, forming interspersed troughs in PC2 accompanied by the accumulation of H3K27me3 as well as stronger compartmental checkering (Fig. 6E). Together, our results show that jointly-derived higher-order components from Hi-C data can provide significant information about long-range interactions relevant to gene expression and epigenetic state dynamics beyond the classic compartment flipping model.

## Discussion

Traditional methods for the analysis of long-range contact frequencies have substantial limitations for integrative analysis because separately applied matrix decomposition methods produce low-dimensional projections onto dissimilar basis vectors. To address this need, we developed *jointly-hic* to normalize and apply PCA to multiple contact frequency maps simultaneously, which projects each dataset onto a common set of basis vectors, allowing for much deeper comparative analyses than previously accessible. Importantly, the *jointly-hic* toolkit implements this methodology while meeting the computational constraints for large-scale integrative studies.

In this study, we demonstrate that joint linear projections capture coherent and biologically meaningful features, and that these features provide consistent interpretations across the stages of an in vitro cardiomyocyte differentiation model system. Critically, we demonstrate that separate linear projections are, in general, significantly misaligned beyond PC1, making them unsuitable for comparison and joint analysis. Further, we show that joint PCA decompositions are robust to sequencing depth variations and background random ligation noise. A key result is that higher-order jointly calculated PC score vectors (i) capture meaningful information beyond the traditional A/B compartment score and (ii) are consistent and comparable across samples, which is not the case for separately calculated projections. While joint PC1 score vectors align quite closely to the traditional A/B compartment vector, we showed that higher-order PCs capture important orthogonal information, including patterns related to chromosome arm alignment and diverse repressed or heterochromatic states. Understanding the mechanistic origins of these higher-order features will help unravel how various cellular and molecular processes and chromatin states influence genome architecture and how such changes drive the evolution of cell state during development.

Signatures of distinct nuclear architectures among broad cell state categories emerged along several components in our atlas-wide joint projections. While the influences of nuclear morphology and sample heterogeneity likely play a role, we found that immune cells, mature tissues, and in vitro ESCs and ESC-derived samples were associated with differences in the epigenetic composition and apparent maturation state of heterochromatin. Immune cells exhibited H3K9me3 with very low levels of H3K27me3 in heterochromatic loci, whereas mature tissues showed a H3K9me3 predominant with mild signal levels of H3K27me3. In contrast, both ESCs and ESC-derived cell types in vitro contained putatively “immature” heterochromatin states characterized by H3K9me2 with minimal presence of the trimethyl mark. This observation, which may be linked to common human embryonic stem cell lines being in a primed, epiblast-like state [53, 54], has direct consequences on genome compartmentalization.

We further identified an intriguing ensemble-wide cluster of loci (EL cluster VII) exhibiting a preponderance of weakly repressive chromatin marks that did not conform neatly to classical heterochromatin definitions, raising the possibility of alternative or transitional heterochromatin states. While EL cluster VI appeared marked primarily by H3K27me3, suggestive of facultative heterochromatin, and VIII was predominantly marked by H3K9me3 in mature cell types, suggestive of constitutive heterochromatin, EL cluster VII contains a mixture of the two marks. One possibility is that these regions represent bivalent states marked by both H3K27me3 and H3K9me3, consistent with recent observations from K9 methyltransferase knockout models, which suggest competitive interplay between these histone modifications [55]. Another possibility is that there is a stronger likelihood of cross-reactivity between ChIP-seq antibodies for their trimethyl target in these lower signal regimes. Further validation, including perturbation experiments of chromatin modifiers, would be invaluable in clarifying the nature of these chromatin states.

One caveat of the *jointly-hic* approach, as an unsupervised method, is that the jointly calculated components are dependent on the input data. For example, when computing joint PCA on only the cardiomyocyte stages, basis vector PC2 correlates with centromere-telomere distance, but when computing joint PCA on the entire atlas of 89 samples, PC4 captures this feature instead. The differences are likely because “Rabl-like” centromere-to-telomere contact frequency patterns in *trans* are more salient in the actively dividing in vitro differentiation samples, and less so in the post-mitotic and more heterogeneous ex vivo tissue samples which make up a large fraction of the atlas. Therefore, in general, when an unsupervised joint PCA is performed in any given study, the biological interpretation of the resulting principal components will need to be determined for that work.

Alternatively, a very large and comprehensive atlas of biosamples could be used to produce a well characterized set of basis vectors that could then be reused by the community at large. New samples can be projected into such a reference latent space by a simple linear transformation. Furthermore, the incremental PCA algorithm used by *jointly-hic* is iterative and stateful, such that new samples may be inexpensively added to an existing atlas of training examples to continually refine the consensus basis vectors [56]. By linear projection, joint PCs produce parametric and biologically meaningful embeddings, which can support a broad range of downstream analyses. For example, one promising avenue would be using joint PCA projections of Hi-C data as harmonized features for training emerging sequence-based deep learning models [57–62], thereby incorporating global long-range 3D genomic data into existing frameworks.

The current study has limitations. While our incremental PCA method is not intrinsically limited by genomic resolution, due to the sparse coverage in *trans* even for some of the most deeply sequenced Hi-C datasets we limited our analyses to a 50-kb bin size, which misses finer compartmental structure [16, 63, 64]. Our reliance on exclusively interchromosomal contact frequency data and a single genomic resolution limits the amount of information gleaned about chromosome organization since *trans* interactions are much less frequent than those occurring in *cis* and therefore also more sensitive to noise. However, the approach does offer advantages, including the ability to obtain genome-wide projections that are automatically harmonized not only across samples but also across chromosomes, and avoiding the need to account for polymeric distance dependences. Focusing on interchromosomal contacts also significantly decouples patterns of compartmentalization and long-range interactions from the patterns of local regulatory interactions and loop extrusion-driven TAD-scale dynamics, even though the latter does exert influence on the former. Future work extending our method to incorporate *cis* data through chromosome-specific joint decomposition will provide even more informative and higher resolution projections which may enhance the sensitivity of case/control studies and perturbation experiments. For example, existing methodologies developed for the differential analysis of the traditional A/B compartment vector may be extended to take higher-order jointly-derived PCs into account [38].

Another potential application of incremental PCA for further development is in the analysis of compartmentalization in single cell Hi-C [65]. In this setting as well as in datasets with fine molecular resolution but limited sequencing depth (e.g. Micro-C), separate PCA projections are strongly influenced by sparsity and noise, necessitating low-resolution or pseudobulk analysis, respectively. Using joint PCA to project a large number of sparse contact matrices into a common low-dimensional space offers a promising complement to such coarse approaches that could help distinguish real variation (between cells or samples) from technical noise.

## Conclusions

In conclusion, our work provides an approach for integrative analysis of genome compartmentalization and patterns of long-range interactions across diverse biological contexts in a manner that is robust to sequencing depth and noise, computationally scalable, and biologically informative. Our findings establish that, when learned jointly, higher-order basis vectors and projections can be used to provide information about genome organization and biological information reflective of cellular identity, chromatin state, and developmental trajectories. By enabling direct comparisons across Hi-C datasets, *jointly-hic* advances our capacity to interpret 3D genome dynamics, laying the groundwork for future integrative studies in development and disease, including perturbation datasets, variant-association studies, and cross-species comparisons.

## Methods

### Joint principal component analysis

The traditional matrix-based approaches to the dimensionality reduction of Hi-C data correspond closely or exactly to PCA and the differences between methods lies largely in the pre-processing of the input matrix. All can be cast as the spectral (eigen) decomposition of (i) a normalized or “observed-over-expected” input contact matrix $X\in {\mathbb{R}}^{n\times n}$, where *n* is the number of genomic bins after coverage-based filtering, following mean-centering [13] or (ii) decomposition of its correlation matrix [12]. If the normalized input matrix is symmetric (${X}^{\top }=X$) and balanced (constant row/column sums), the former approach produces the same vectors as a conventional PCA, up to a scale factor, which can be expressed as the eigenvectors of the covariance matrix $cov(X)=\frac{1}{n-1}{\left(X-\overline{X}\right)}^{\top }\left(X-\overline{X}\right)$. The latter approach corresponds to a correlation-based PCA, which is equivalent to applying an additional z-score transformation to the features of $X$ before performing conventional PCA. In general, both types of PCA are normally computed by performing a singular value decomposition (SVD) of the mean-centered input matrix, where right singular vectors correspond to principal components.

To perform a joint PCA factorization on an arbitrary number of contact frequency profiles (observations) over many input contact matrices, *jointly-hic* uses the IncrementalPCA estimator from *scikit-learn* [40], which performs an iteratively-updated SVD in mini batches, returning the most significant singular vectors [19, 56]. This algorithm has constant memory complexity, on the order of the batch size times the number of features. Altogether, the *jointly-hic* pipeline consists of the following steps:

1. Genome-wide balanced contact frequency matrices are loaded from multiresolution cooler [66] (mcool) files at the desired genomic resolution. Only autosomes (chromosomes 1–22) were included for the analyses in this study.
2. Intrachromosomal contacts (*cis* regions) are removed and replaced with randomly sampled *trans* pixels from the same row/column, as in [67].
3. Blowout pixels, defined as above the 99.5th percentile threshold, were clipped. Pixels below the first percentile were set to 0 to remove additional poor coverage bins.
4. Contact matrices are rebalanced so that all rows and columns sum to 1.
5. Genomic bins that get masked in any of the input samples are combined into a union list and pre-processed input matrices are saved to disk for further stages.
6. For each pre-processed input matrix, incremental PCA is run, updating in chunks of 10,000 rows per iteration via the `partial_fit` method. After each input matrix is exhausted, the next is loaded.
7. Once the final joint PCA model is fit, a second pass through the data is performed to transform the input matrices into PC score vectors (projections).

In general it is challenging to determine purely technical batch effects to correct for a priori. However, we noticed that increased background ligation noise in our simulations tended to decrease the overall dispersion of the sample-loci from the origin for a given sample isotropically. To correct for such sample-to-sample differences in background noise levels, we divide the matrix of PC score vectors derived from each sample by the Frobenius (matrix) norm for that sample, which normalizes how “spread out” each sample’s point cloud is around the origin. The final PC score vectors are rescaled to the global norm of the original projections.

We note that a similar workflow with different pre-processing steps can be used to jointly embed *intra*-chromosomal Hi-C maps. For pre-processing, the power-law distance decay of contact frequency—by far the dominant feature of such maps—must be corrected for. Depending on how such “nuisance” *cis* features are modeled, the resulting observed-over-expected maps may capture residual chromosome-specific phenomena to different degrees (e.g., chromosome arm alignment, loop extrusion features) and therefore pre-processing intrachromosomal maps for large-scale joint analyses should be done with care [13, 15, 38, 68]. Most importantly, intrachromosomal joint projections, while consistent across input samples, will be different for each genomic unit analyzed (chromosome, chromosome arm segment, etc.), which will introduce additional challenges in making correspondences between results obtained from different units (e.g., cluster labels).

### HCT116 downsampling and noise injection

We verified the reproducibility of projections from *jointly-hic* using the unsynchronized, untreated RAD21-Auxin-inducible degron (AID) HCT116 dataset from [44]. The pseudodiploid colorectal cancer cell line was processed using *distiller-nf* and subjected to downsampling and synthetic injection of random ligations. Non-nuclear, sex chromosomes, and three large autosomal translocations were excluded as previously described [21]. Downsampling was performed using *cooltools sample* [15]. The number of removed counts was replaced with randomly generated ligation pairs drawn uniformly across the genome to restore total read counts. Using *jointly-hic*, we computed joint PCA projections for the original, downsampled, and downsampled with artificial noise samples.

### Cardiomyocyte differentiation separate and joint PCA

We applied PCA separately to each contact matrix, generating sPCA projections for each sample. Independently, we also applied the full *jointly-hic* processing pipeline to generate jPCA projections. Genomic features including GC content and centromere-telomere distance computed at the same loci were merged with the sPCA and jPCA projections with the help of *bioframe* v0.8.0 [67]. Due to the arbitrary algebraic sign of sPC score vectors from stage to stage, we calculated absolute values of Pearson correlation coefficients between all sPC and jPC score vectors within and across stages. Pairwise angles between sPC2 and sPC3 score vectors in different differentiation stages was computed by calculating cosine similarities and converting to the corresponding acute angle in degrees.

### Benchmarking *Jointly-hic* using cardiomyocyte datasets

To examine the performance of *jointly-hic* with respect to variation in the resolution, we systematically benchmarked the software using the datasets for six stages of cardiomyocyte differentiation. We applied the full *jointly-hic* pipeline to these six stages at resolutions of: 25, 50, 100, 250 and 500 thousand base pairs while monitoring the runtime, CPU utilization and memory usage. We performed this analysis on a server with 64 real cores (AMD EPYC 7763), 512 GB of RAM, and an SSD-backed network file system. We plotted bar plots of runtime and maximum memory usage to run the pipeline.

Next, we sought to examine how bin-level principal components vary with respect to analysis resolution. We matched bins at corresponding resolutions (e.g. chr1:125,000–150,000 matches chr1:100,000–200,000) and duplicated (upsampled) the lower resolution values to match. Then we computed the Pearson correlation of the sample-bin scores. To visualize this, we chose 100 kb resolution as a reference and computed the correlation of bins compared to 100 kb resolution. We plotted bar plots of the Pearson correlation for components one through 6 compared.

### Biosample atlas data curation

We curated an atlas comprising 90 human Hi-C datasets. We included 73 Hi-C samples from the ENCODE data portal, containing greater than 1 billion ligation pairs and greater than 200 million non-negative bins, from ex vivo and primary culture biosamples. We excluded immortalized and cancer cell lines and samples with abnormal karyotype or structural variants. We also included human in vitro differentiation model systems representing the heart [42], pancreas [41] and liver (4DN consortium) from published studies and the 4DN consortium. After applying joint PCA, we excluded one sample (ENCODE accession ENCSR797MWY, aorta tissue from a 41 year old female) from all further analysis. Its PC projection differed substantially from all others, and upon visual inspection of the contact map, this sample showed pervasive and highly unusual chromosome-level fluctuations in contact frequency in *trans*, suggestive of extensive aneuploidy or some perhaps some other phenomenon, such as homologous chromosome pairing recently reported in adult aortic endothelial cells [69]. Sample metadata including experiment and file accessions and biosample information are available in Additional file 2: Table S2. In total, we curated an atlas of 90 Hi-C datasets and kept 89 for all analysis in this study.

We prepared Hi-C datasets in mcool files for analysis, visualization and processing using *jointly-hic* (version 1.0.0)*.* ENCODE samples were downloaded from the ENCODE portal as hic format files. For these samples, read quality-control, alignment to the hg38 genome, filtering, and conversion to contact frequency matrices were already performed with the ENCODE implementation (github.com/ENCODE-DCC/hic-pipeline) of the Juicer pipeline [70]. The hic format files were converted to the cooler format using *hictk* (version 0.0.10) at 1000 base pair resolution [71]. We used *cooler * [66] to “zoomify” and re-balance these to multi-resolution cooler (mcool) files. Sequencing read data from differentiation models were downloaded as FASTQ files from Gene Expression Omnibus with the accession GSE116862 for the cardiac and GSE210524 for the pancreas systems. Data for the hepatocyte differentiation model was obtained directly from collaborators and is available via the 4DN data portal under accessions provided in Additional file 2: Table S2. Raw sequencing reads were processed using *distiller-nf* (version 0.3.4) through read quality-control, alignment to the hg38 genome, filtering, and conversion to contact frequency matrices (github.com/open2c/distiller-nf). To complement the atlas of Hi-C datasets within a broader functional context, we curated collections of histone ChIP-seq, transcription factor (TF) ChIP-seq, and ATAC-seq. We queried ENCODE for all matching files, choosing data processed by ENCODE4, aligned to hg38, with the status “released,” and a biosample name matching one of the Hi-C atlas biosamples. In total, we collected 1,410 Histone ChIP-seq tracks (Additional file 2: Table S3), 321 TF ChIP-seq tracks (Additional file 2: Table S4), and 185 ATAC-seq tracks (Additional file 2: Table S5). Each signal track was downloaded from the ENCODE portal in BigWig format [72]. We integrated spatial landmark data from the 4DN data portal for DamID-seq targeting AP3D1 and LMNB1, and TSA-seq targeting LMNB1, SON, POLR1E, NIFK, and CENPB. The tracks and accessions are available in Additional file 2: Table S6. We downloaded processed BigWig files for LMNB1 ChIP-seq and H3K9me2 ChIP-seq from GEO, with the accessions in Additional file 2: Table S7 [49]. Genome browser visualizations were generated using HiGlass [73] and *higlass-python* (github.com/higlass/higlass-python).

### PC score vectors and signal track aggregation

Following joint PCA, metadata tables were generated containing experiment metadata about biosample names, accessions, and mappings between file names and experiments in YAML format. Using *jointly-hic*, we created a custom HDF5-based database called a “JointDB” containing all jPCA projections from Hi-C at 50-kb resolution and corresponding epigenetic signal data from BigWig files of ChIP-seq, ATAC-seq, and other epigenetic profiling assays aggregated at the same resolution, as well as experiment metadata from all included samples and tracks. The JointDB can be queried as pandas dataframes using the *jointly-hic* software.

For each cell state category, we collated 50 kb bin-level PC scores with matching signal tracks from the same cell state category. These tracks included GC content and genomic distance from centromere. We calculated Spearman correlation coefficients of the 50-kb signal tracks separately for each sample PC score vector by each biosample-matching matching track, and displayed the mean correlation coefficient in bubble plots sized by the number of tracks contributing to the calculation. We also collated all 50 kb bin-level ChIP-seq profiles of H3K27me3 and H3K9me3 for each cell state category, mean-centered and scaled them by standard deviation, and generated violin plots to show the scaled signal distribution among the three categories. Subcompartment labels for GM12878 were lifted over from hg19 to hg38 using UCSC liftover and upsampled to 50-kb resolution.

### Atlas sample-level overview

We generated an overview of the Hi-C atlas by analyzing the full sets of genomic bin-level PC scores for each sample. We concatenated the first 4–32 PC score vectors from the *jointly-hic* projection for each of the 89 biosamples and applied a secondary PCA with two output components to visualize sample-level similarities. These secondary projection plots remained visually stable after including 5 or more top PC score vectors. We also applied hierarchical Ward clustering to the sample-level flattened feature vectors consisting of the first 12 PC score vectors and computed the euclidean distance map between them using *scipy* [74] and *fastcluster* [75].

### Atlas locus and sample-locus cluster analysis

For sample-locus clustering, we applied Leiden clustering (leidenalg v.0.10.2) to all sample-locus projections simultaneously over a range of resolutions including 0.1, 0.2, 0.3, 0.5, 0.8 and 1.0 and using 500 as the nearest neighbor cutoff [76]. We selected resolution parameter value 0.5, corresponding to 11 clusters, for further analysis. For ensemble-wide locus clustering, we applied K-means clustering using *scikit-learn* to the locus-level projections, i.e. to the full set of sample-level projections for each locus, where each input vector corresponds to the concatenation of 32 PC score vectors from each sample. K-means was computed for a range of k including 5, 6, 7, 8, 9, 10, 12, 15, 20. UMAP was performed using umap-learn v0.5.6 [77].

### Cardiomyocyte differentiation RNA-seq analysis

FASTQ files of RNA-seq data from six time points, each represented by two replicates: day 0, day 2, day 5, day 7, day 15, and day 80, corresponding to the Hi-C data, were downloaded from SRA. We used Salmon v1.10.3 for transcript-level quantification against the Gencode v46 transcripts annotations and GRCh38 primary assembly with the “validateMappings” and “gcBias” flags [78]. Then we used tximport v.1.30.0 to aggregate transcript abundances to the gene level [79]. A sample metadata table was generated to map each sample to its corresponding time point and experimental stage, which enabled a time-based design in the subsequent differential expression analysis. We applied PyDESeq2 v0.5.1 to compute differential gene expression statistics between consecutive stages [80]. We overlapped the genomic coordinates of increasing and decreasing differentially expressed gene TSSs with atlas bins and their jPCA scores at all developmental stages with *bioframe*. For each sequential stage transition, we used *statsmodels* [81] to fit linear models to predict log fold changes in RNA-seq TPM using differences in PC scores as regressors.

### Cardiomyocyte differentiation ATAC-seq analysis

For the cardiomyocyte time-course experiments, we downloaded ATAC-seq sequence data for the six developmental stages, each with two replicates, corresponding to the RNA-seq and Hi-C experiments. We processed the data using the ENCODE ATAC-seq pipeline (github.com/ENCODE-DCC/atac-seq-pipeline). We generated signal BigWigs directly from the filtered alignment files. To correct for transposase cutting biases, we first used the alignmentSieve utility (part of the deepTools suite, version 3.5.5) with the “ATACshift” parameter to adjust each BAM file [82]. The shifted BAM files were then sorted and indexed using samtools v1.13 [83]. Following sorting, coverage tracks were created with bamCoverage (deepTools) using a 1 bp bin size, with normalized coverage by the read-per-genome-coverage (RPGC) method, exact scaling, and excluding duplicate reads. Each coverage track was stored in BigWig format, and also averaged over 50-kb tiling genomic bins and added to the JointDB database. We quantified ATAC-seq coverage at ENCODE candidate cis-regulatory elements (cCREs, version 4 [35]) through a read count based approach. First, cCRE regions were defined in a featureCounts SAF (Simple Annotation Format) file containing genomic coordinates. We then used featureCounts (v1.5.3) in pair-end mode to count reads overlapping the cCRE intervals across all replicate BAM files [84]. We applied PyDESeq2 v0.5.1 to compute differential accessibility statistics between consecutive stages [80].

### Cell state category contact maps and saddle plots

To visualize differences between the cell state category groups, we created “mega-maps” by merging all relevant contact frequency matrices using the *cooler merge* command, followed by *cooler zoomify* to generate multi-resolution, balanced matrices. These aggregated contact maps capture an averaged contact pattern representative of each class. Using *cooltools*, we calculated per-chromosome expected contact frequencies (*cis* and *trans*) as input for saddle plot generation. The saddle function in cooltools was then applied to calculate saddle plot matrices from each individual sample contact matrix. Continuous saddle strength metrics were calculated from each saddle plot matrix as previously described, providing a ratio of intra-compartment interactions to inter-compartment interactions over successive quantile bins along the eigenvector. The average and standard error envelope of these curves for each cell state category were plotted. A slightly modified version of the *cooltools* saddle function was used to calculate “discrete” saddle plots over EL cluster categories.

### Locus-level heatmap, projection scatter plots, and overlays

The locus-level heatmap was generated with matplotlib from 50-kb PC score vectors and signal tracks from the JointDB using custom scripts. Rows representing score vectors and signal tracks were annotated grouped based on cell state category and ordered within each group. Columns representing 50-kb bins were grouped by EL cluster assignment. The EL clusters themselves were ordered and numbered by mean GC content, and within each group the columns were ordered by genomic distance from the centromere.

To avoid issues with overplotting of very dense scatter plots and support the overlaying of various quantitative and categorical variables associated with sample-loci in our various projection visualizations, we used the the *matplotlib* [85] extension of the *datashader* package [86] (datashader.mpl_ext.dsshow). This tool allowed for accurate rendering of two-dimensional point density plots and other aggregates of our PCA projections and UMAP sample-locus embeddings as well as gene-level scatter plots and volcano plots. With dsshow, point counts or alternate aggregations over points, such as mean values of a signal track, are generated over pre-defined grids in 2D cartesian space and rendered as raster images with a user-defined color map. Categorical aggregations are performed by color-encoding the categorical labels of points, quantitatively scaling the aggregates for each category using transparency, and compositing the resulting color channels. We also implemented a plotting tool to generate radial “star coordinates” plots from a given joint PC subspace using matplotlib’s scatter function for vector-based rendering or dsshow for raster-based rendering (https://gist.github.com/nvictus/f90b32503a3da126e2c72fda13a7119c). These plots project n-dimensional data points onto a two-dimensional circle with each dimension axis separated by equal angles around the origin.

## Supplementary Information

Additional file 1.

Additional file 2.

## Data Availability

The *jointly-hic* software package is open source, licensed under GPL-3.0, and available at github.com/abdenlab/jointly-hic [43]. The version used in this study is v1.0.0 and available at https://zenodo.org/records/15198530 [87]. Metadata and accession numbers for the datasets used and/or analyzed in this study are included in Additional file 2: Tables S1-S6. Published Hi-C, ChIP-seq, and ATAC-seq data can be found on the ENCODE and 4DN data portals [37, 88] at the accessions indicated. Additional published Hi-C data was obtained from GEO repositories GSE210524 [89] and GSE116862 [90]. Additional published ChIP-seq data was obtained from GEO repository GSE155244 [91].
