# Supplementary material for: Jointly-hic: joint decomposition of contact frequency maps captures salient features of genome architecture across tissues and development
Source: Genome Biol. 2026 May 2;27:205. doi: 10.1186/s13059-026-04067-1 (PMC13285045; doi:10.1186/s13059-026-04067-1)
Supplement: Supplementary file 1 — Additional file 1. [file 13059_2026_4067_MOESM1_ESM.pdf]

Fig. S1

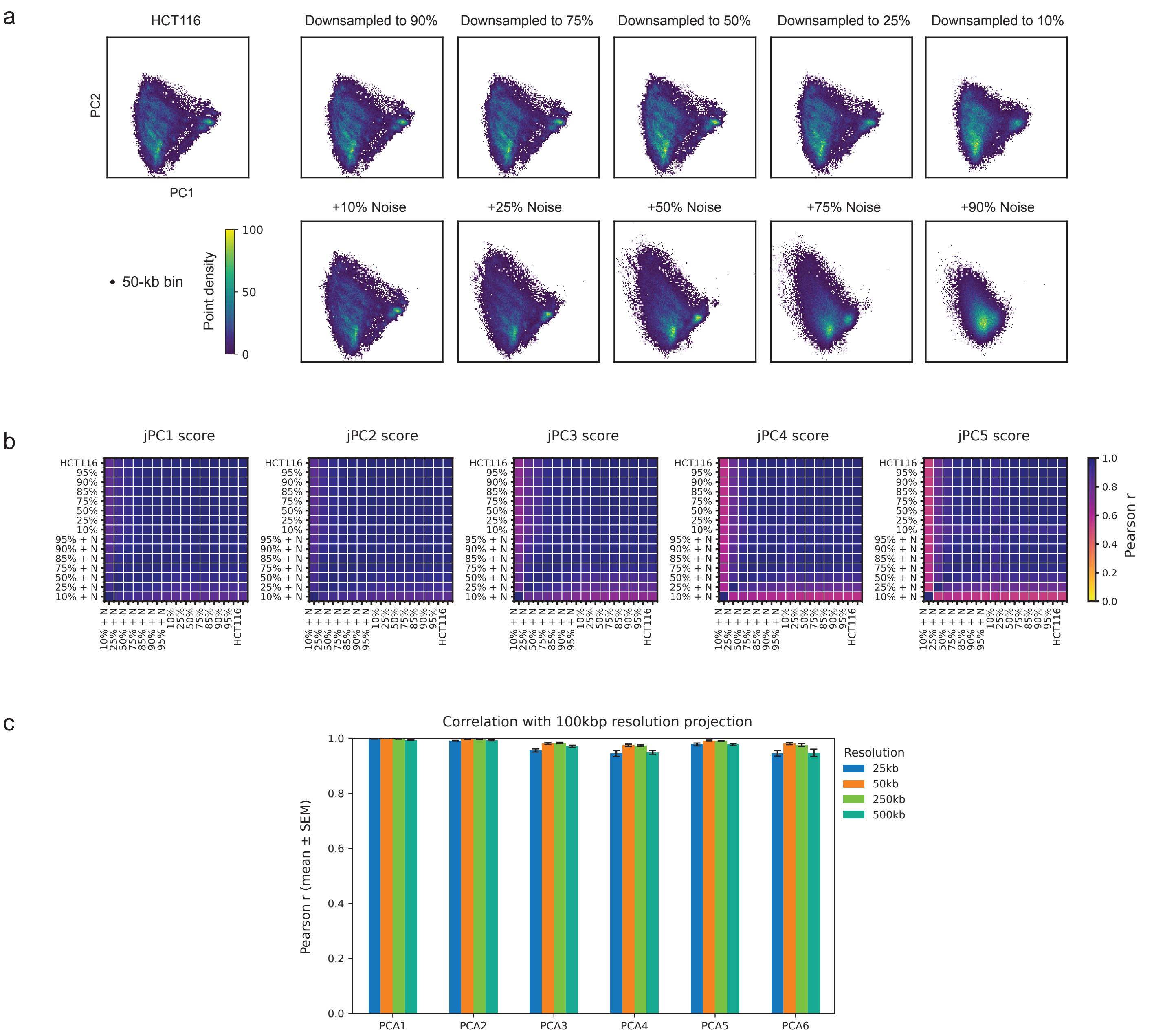

Fig S1. Joint PCA is robust to sequencing depth, noise and resolution. (A) Scatter plots of jPC1 vs jPC2 scores for joint PCA of a 50-kb resolution HCT116 contact map before and after progressive downsampling of read pairs (top row) and downsampling with replenishment using simulated random ligation pairs up to the original read depth (bottom row). Points represent 50-kb genomic loci. (B) Heatmaps of Pearson correlation coefficients between all same-ranked PC score vectors derived from the various perturbed HCT116 datasets shown in (A) for PCs 1-5. (C) Bar plots of mean Pearson correlation between each of PC score vectors 1 through 6 from six samples, calculated from input data at four different resolutions, compared to a 100-kb resolution reference projection. Error bars represent the standard error.

Fig. S2

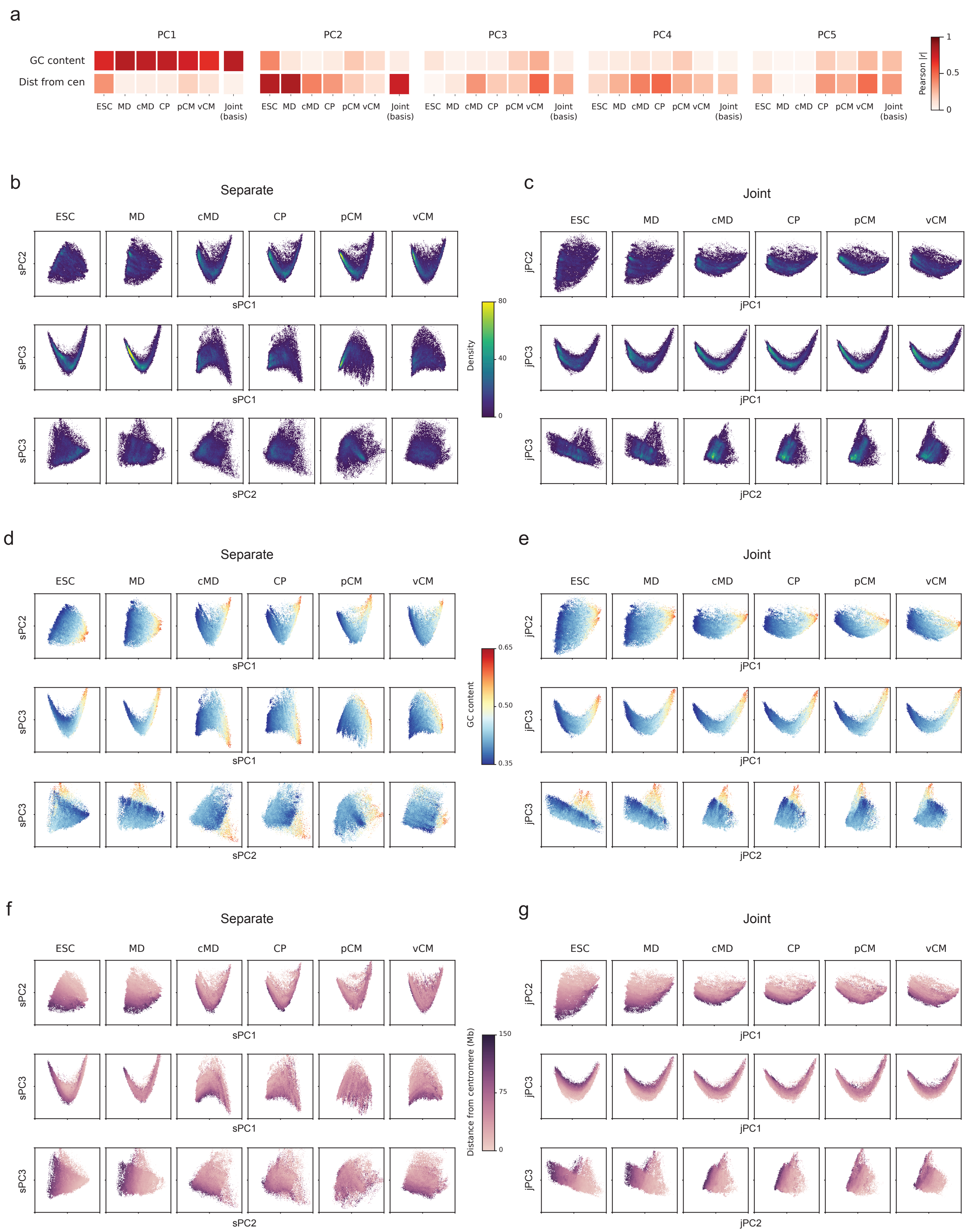
a
PC1
PC2
PC3
PC4
PC5
GC content
Dist from cen
ESC MD cMD CP pCM vCM Joint (basis)
Pearson |r|
1
0.5
0
b
Separate
ESC MD cMD CP pCM vCM
sPC2
sPC1
sPC3
sPC1
sPC3
sPC2
Density
80
40
0
c
Joint
ESC MD cMD CP pCM vCM
jPC2
jPC1
jPC3
jPC1
jPC3
jPC2
d
Separate
e
Joint
GC content
0.65
0.50
0.35
f
Separate
g
Joint
Distance from centromere (Mb)
150
75
0

Fig S2. Misalignment of sPCA basis vectors across samples. (A) Heatmaps of absolute Pearson correlation coefficients between 50-kb tracks of GC content (top row) and genomic distance from the centromere (bottom row) with sPC vectors from each stage as well as jPC basis vectors. (B) PC score scatter plots for samples representing six successive in vitro cardiomyocyte differentiation stages, derived by separate PCA (sPCs). The first row shows sPC1 vs sPC2, the second row shows sPC1 vs sPC3, and the third row shows sPC2 vs sPC3. Each point represents a 50-kb bin. (C) PC score scatter plots for samples representing six successive in vitro cardiomyocyte differentiation stages, derived by joint PCA (jPCs). The first row shows jPC1 vs jPC2, the second row shows jPC1 vs jPC3, and the third row shows jPC2 vs jPC3. Each point represents a 50-kb bin. (D) The same PC score scatter plots as (B), colored by GC content. (E) The same PC score scatter plots as (C), colored by GC content. (F) The same PC score scatter plots as (B), colored by genomic distance from the centromere. (G) The same PC score scatter plots as (C), colored by genomic distance from the centromere.

Fig. S3

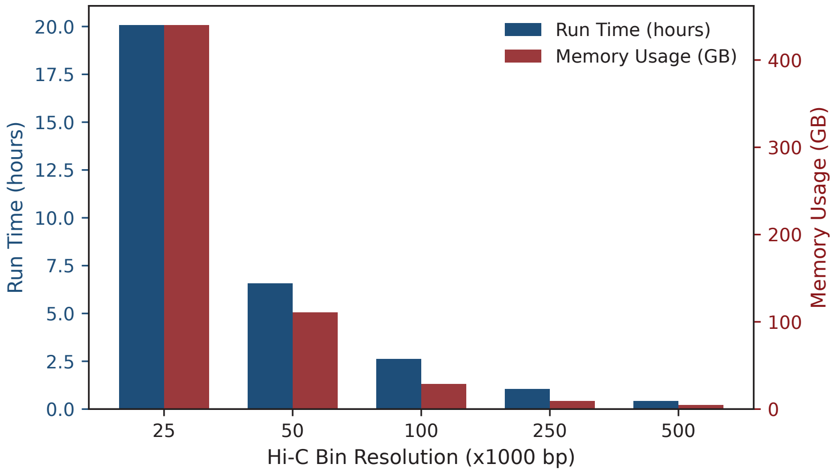

Fig S3. Runtime characteristics of jointly-hic. Runtime and memory usage statistics for 6-stage cardiomyocyte ensemble. Jointly-hic was applied at 25, 50, 100, 250, and 500 kb resolution. Blue (left axis) shows runtime in hours. Red (right axis) shows maximum memory usage.

Fig. S4

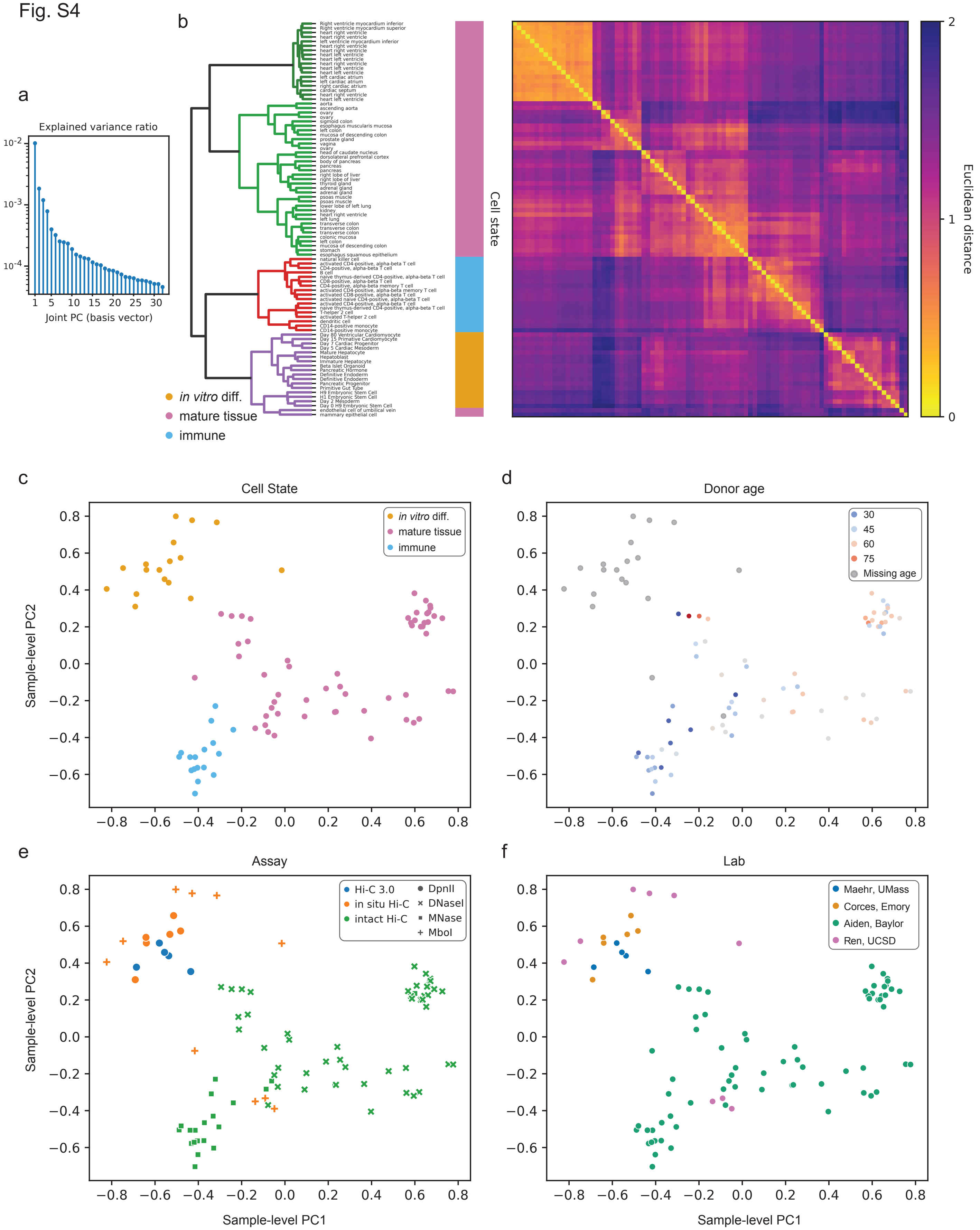

a
Explained variance ratio
Joint PC (basis vector)
b
in vitro diff.
mature tissue
immune
Cell state
Euclidean distance
c
Cell State
d
Donor age
30
45
60
75
Missing age
e
Assay
Hi-C 3.0
in situ Hi-C
intact Hi-C
DpnII
DNaseI
MNase
MboI
f
Lab
Maehr, UMass
Corces, Emory
Aiden, Baylor
Ren, UCSD
Sample-level PC1
Sample-level PC2

Fig S4. Sample-level hierarchical clustering and similarity of genome-wide contact frequency profiles as measured by joint PCA. (A) Explained variance ratios of atlas PCs. (B) Ward clustering dendrogram and Euclidean distance heatmap between sample-level secondary PC projections of the first 12 sample-locus PC score vectors. (C-F) Sample-level PCA plot as in Figure 3B colored by sample covariates including: (C) assigned cell state category, (D) donor age, (E) Hi-C assay protocol, and (F) production lab.

Fig. S5

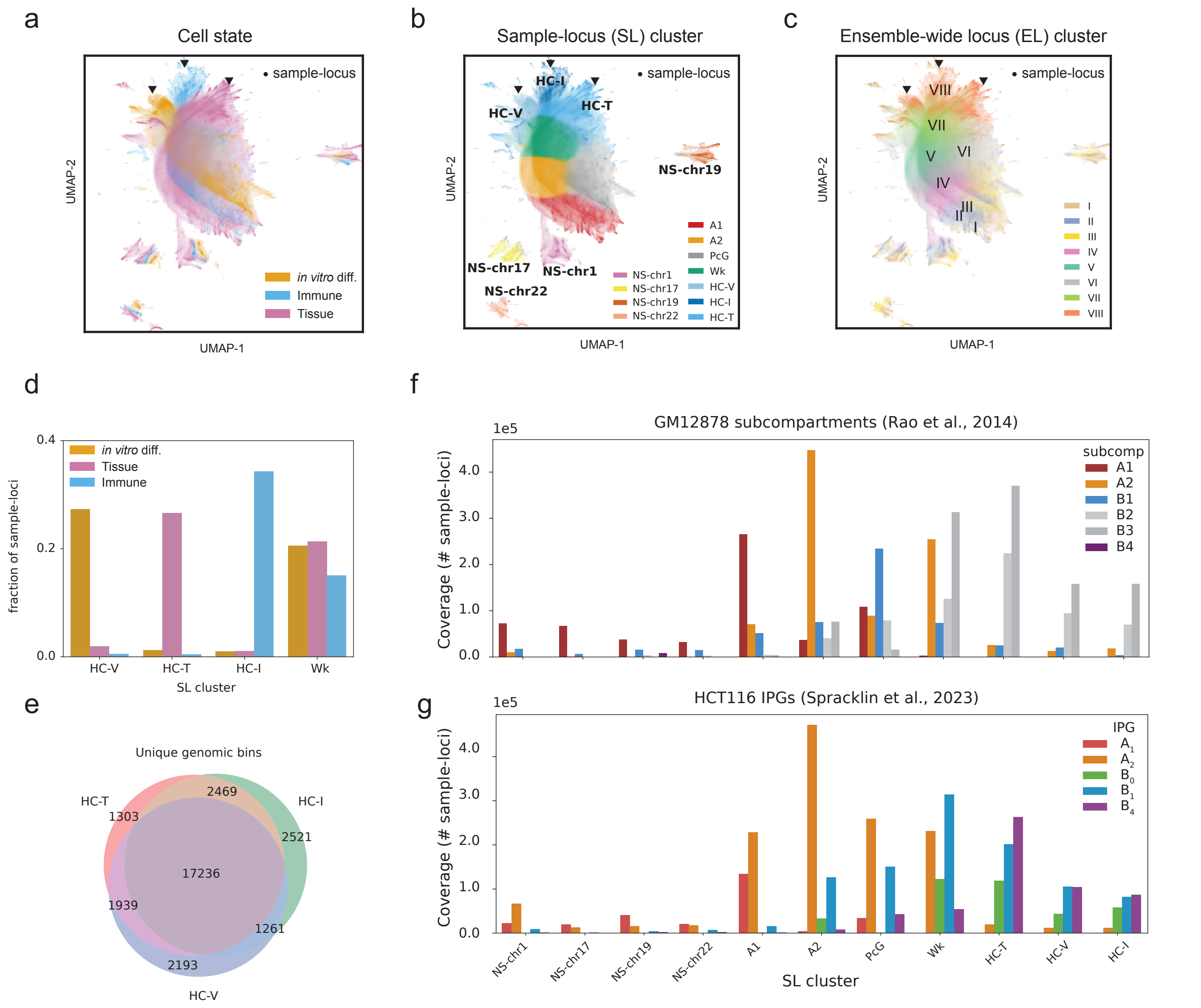

Fig S5. UMAP embeddings of the interaction profile atlas and characterization of SL clusters. (A) Visualization of the UMAP embedding derived from the full set of 32 PC scores for each 50-kb sample-locus colored by the cell state category of the corresponding sample. The locations of SL clusters HC-V, HC-I, and HC-T are shown in all three panels with black triangles. (B) Same as (A) but colored by sample-locus (SL) cluster label. The locations of SL clusters HC-V, HC-I, and HC-T are shown in all three panels with black triangles. The nuclear speckle "island" clusters are also labeled. (C) Same as (A) but colored by the ensemble-wide locus (EL) cluster label of the corresponding genomic bin. The locations of SL clusters HC-V, HC-I, and HC-T are shown in all three panels with black triangles. (D) Bar plots showing fractions of all sample-loci within each cell state category assigned to the SL clusters HC-V, HC-T, HC-I, and Wk. (E) Venn diagram showing the overlap of the unique 50-kb genomic bins assigned to SL clusters HC-V, HC-T, and HC-I. (F) Bar

chart displaying the distribution of corresponding GM12878 subcompartment labels among sample-loci in each SL cluster. (G) Bar chart displaying the distribution of corresponding HCT116 IPG labels among sample-loci in each SL cluster.

Fig. S6

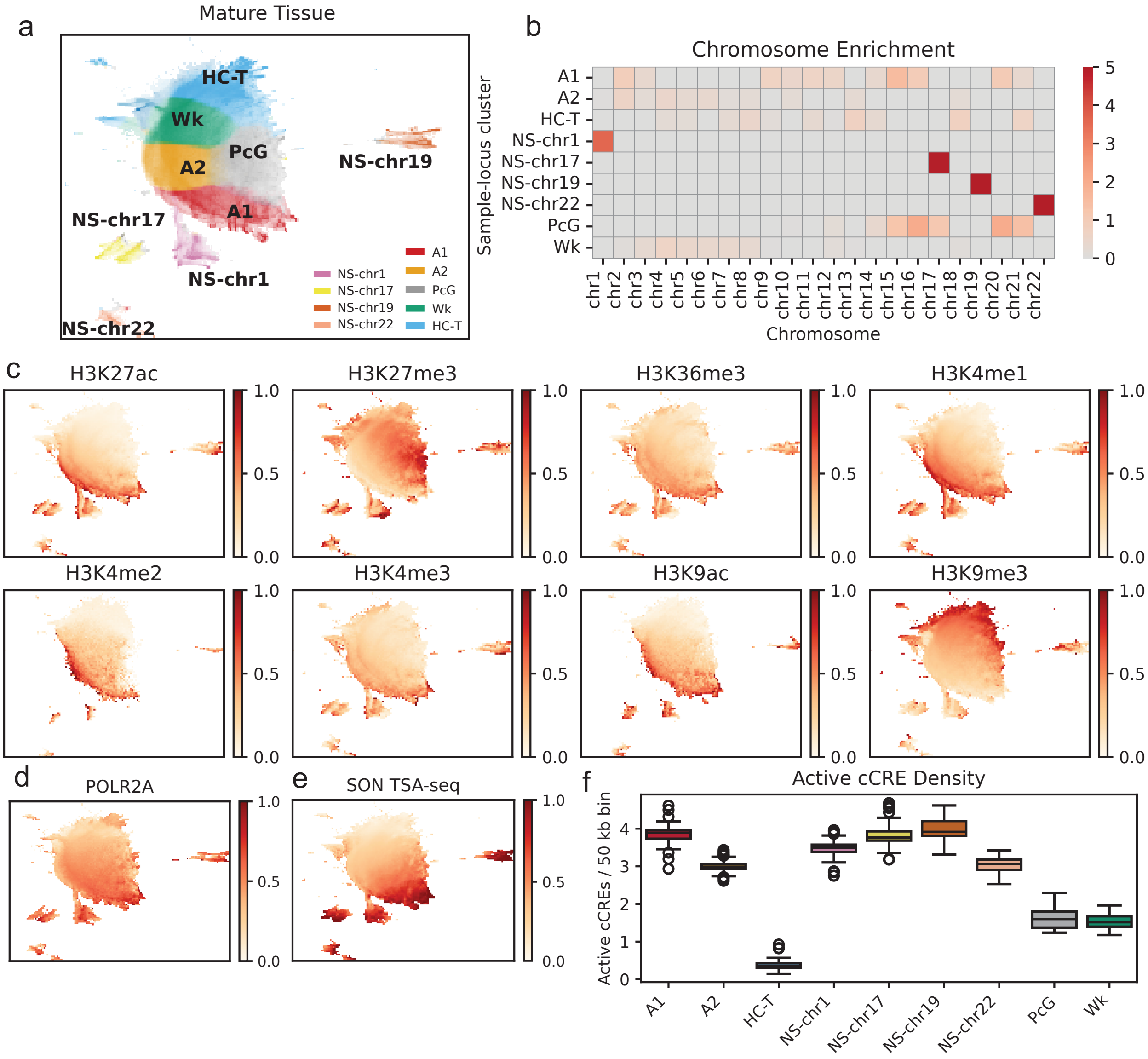

Fig S6. Four sample-locus clusters correspond to nuclear speckle-associated regions on specific chromosomes. (A) Visualization of a UMAP embedding all genomic bins from 55 mature tissue samples colored by sample-locus cluster label. NS-chr1, NS-chr17, NS-chr19, and NS-chr22 correspond to chromosome-specific nuclear speckle "islands". (B) Chromosome-level enrichment for sample-locus clusters in mature tissue samples (observed / expected) shows that speckle-associated island clusters are enriched for a specific chromosome. (C) Mean ChIP-seq signal quantile from matched biosamples on ENCODE overlaid over UMAP embeddings. (D-E) POLR2A ChIP-seq (D) and SON TSA-seq (E) mean signal quantiles overlaid against all bin UMAP scatter plots show enrichment in A1 and speckle island clusters. (F) Box and whisker plot of active cCREs per genomic bin across matching mature tissue biosamples for each sample-locus cluster.

Fig. S7

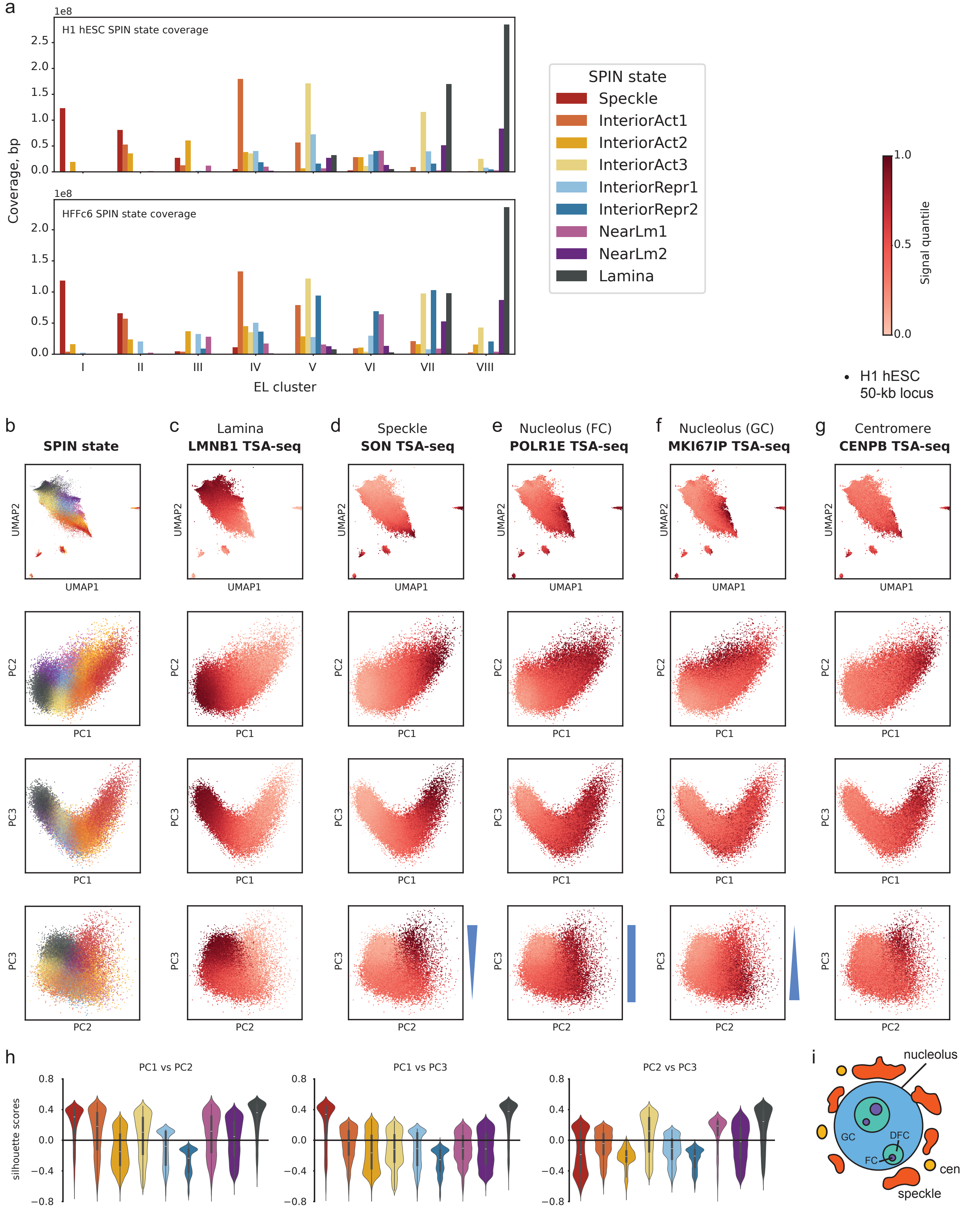
a
H1 hESC SPIN state coverage
HFFc6 SPIN state coverage
Coverage, bp
1e8
EL cluster
I
II
III
IV
V
VI
VII
VIII
SPIN state
Speckle
InteriorAct1
InteriorAct2
InteriorAct3
InteriorRepr1
InteriorRepr2
NearLm1
NearLm2
Lamina
Signal quantile
H1 hESC
50-kb locus
b
SPIN state
c
Lamina
LMNB1 TSA-seq
d
Speckle
SON TSA-seq
e
Nucleolus (FC)
POLR1E TSA-seq
f
Nucleolus (GC)
MKI67IP TSA-seq
g
Centromere
CENPB TSA-seq
UMAP1
UMAP2
PC1
PC2
PC3
h
PC1 vs PC2
PC1 vs PC3
PC2 vs PC3
silhouette scores
i
nucleolus
GC
DFC
FC
cen
speckle

Fig S7. The spatial proximity to nuclear landmarks as measured by TSA-seq is explained by multiple PC basis vectors. (A) Genomic coverage of SPIN state annotations from (top) H1 hESC and (bottom) HFFc6 cells overlapping ensemble-wide locus (EL) clusters. (B) Scatter plot visualizations of H1 hESC 50-kb bin PC scores, colored by H1 SPIN states. Top: UMAP embedding; second row: PC1 vs PC2; third row: PC1 vs PC3; fourth row: PC2 vs PC3. (C-G) Same scatter plots as (B) but colored by signal quantile of TSA-seq targeting marker proteins associated with: (C) the nuclear lamina (LMNB1), (D) nuclear speckles (SON), (E) nucleolar follicular centers (POLR1E), (F) nucleolar granular component (MKI67IP), and (G) centromeres (CENPB) in H1 cells. Opposite gradients of SON and MKI67IP TSA-seq signals along PC3 are highlighted with blue triangles on the right of the PC2-PC3 plots. A blue rectangle next to the corresponding POLR1E plot represents flat POLR1E signal enrichment along PC3. (H) Violin plots of SPIN label silhouette scores calculated on the two-dimensional points in PC score scatter plots from (B) quantify how well the PC projections cluster the SPIN labels. (I) Cartoon of the nucleolus (blue) and surrounding landmarks, nuclear speckles (red) and centromeres (yellow) in H1 cells. GC: granular component, DFC: dense follicular center, FC: follicular center, cen: centromere.

Fig. S8

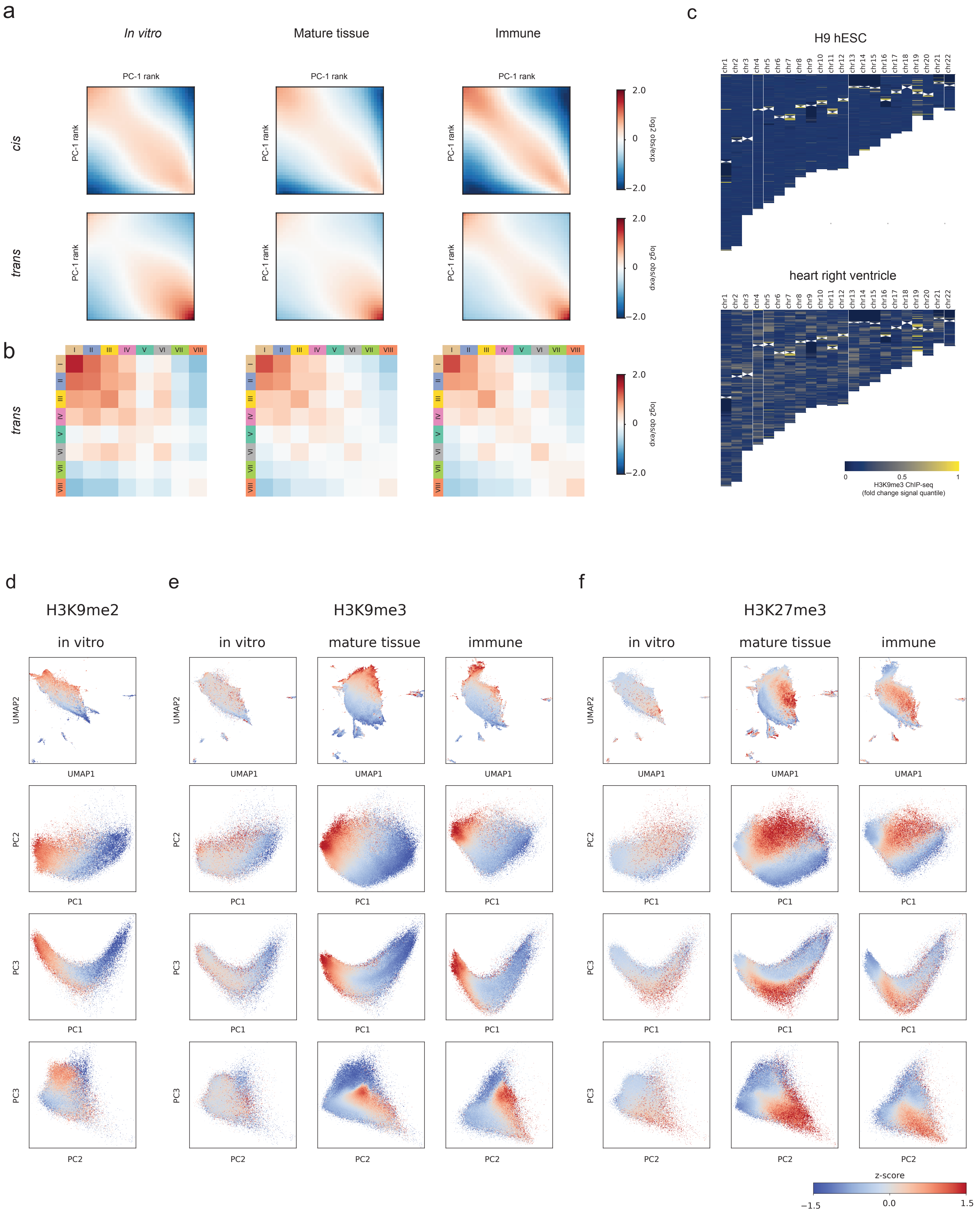

a
In vitro
Mature tissue
Immune
PC-1 rank
cis
trans
2.0
0
−2.0
log2 obs/exp
b
I
II
III
IV
V
VI
VII
VIII
c
H9 hESC
heart right ventricle
chr1
chr2
chr3
chr4
chr5
chr6
chr7
chr8
chr9
chr10
chr11
chr12
chr13
chr14
chr15
chr16
chr17
chr18
chr19
chr20
chr21
chr22
0
0.5
1
H3K9me3 ChIP-seq
(fold change signal quantile)
d
H3K9me2
e
H3K9me3
f
H3K27me3
in vitro
mature tissue
immune
UMAP1
UMAP2
PC1
PC2
PC3
z-score
−1.5
0.0
1.5

Fig S8. Saddle plots and PC score visualizations of repressive histone marks within each cell state category. (A) Continuous saddle plots for PC-1 rank in cis and trans for each cell state category. (B) Discrete saddle plots of average observed over expected contact frequency between EL clusters. (C) Ideogram heatmaps showing the intensity and genomic distribution of broad, interspersed H3K9me3 ChIP-seq signal in H9 hESC cells (top) and heart right ventricle cells (bottom) at 50-kb resolution as representative in vitro differentiation and mature tissue samples. (D) UMAP (top) and PC score (bottom) scatter plots of sample-locus long-range interactions colored by H3K9me2 signal z-score from matched in vitro differentiation biosamples. Matching datasets for other cell state categories were not available. (E) UMAP (top) and PC score (bottom) scatter plots of sample-locus long-range interactions colored by H3K9me3 signal z-score from matched biosamples for each of the three cell state categories (columns). (F) UMAP (top) and PC score (bottom) scatter plots of sample-locus long-range interactions colored by H3K27me3 signal z-score from matched biosamples for each of the three cell state categories (columns).

Fig. S9

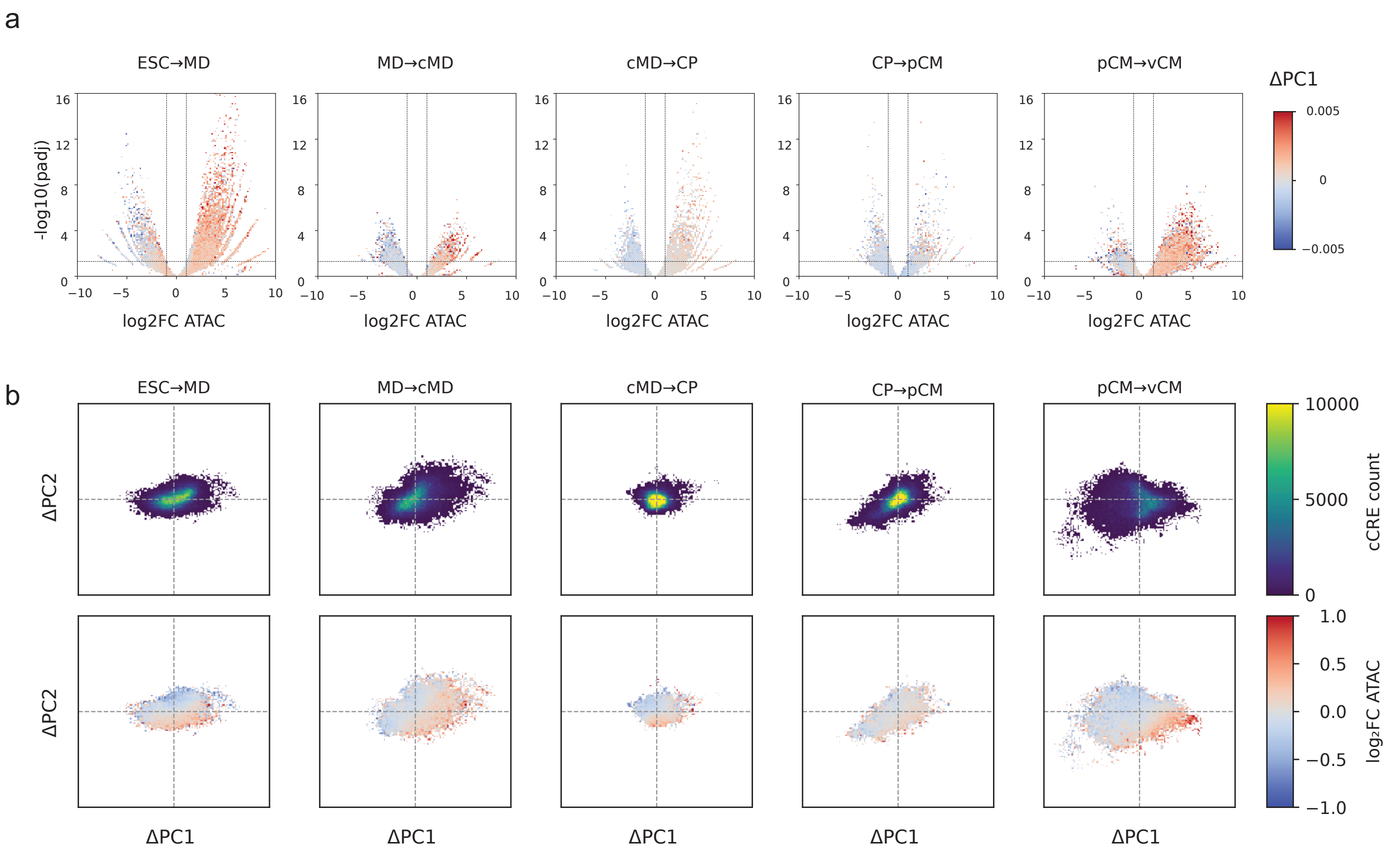

Fig S9. Relationship between accessibility of candidate cis-regulatory elements and changes in PC1 and PC2 during in vitro differentiation. (A) Volcano plots of differential chromatin accessibility at ENCODE cCREs between consecutive stages of cardiomyocyte differentiation, colored by change in PC1 score. **(B)** Scatter plots of change in PC1 score vs change in PC2 score at ENCODE cCREs, colored by point density (top) and log2 fold change ATAC-seq signal (bottom).
